# NFAT1 Orchestrates Spinal Microglial Transcription and Promotes Microglial Proliferation via c‐MYC Contributing to Nerve Injury‐Induced Neuropathic Pain

**DOI:** 10.1002/advs.202201300

**Published:** 2022-07-27

**Authors:** Bao‐Chun Jiang, Ting‐Yu Ding, Chang‐Yun Guo, Xue‐Hui Bai, De‐Li Cao, Xiao‐Bo Wu, Wei‐Lin Sha, Ming Jiang, Long‐Jun Wu, Yong‐Jing Gao

**Affiliations:** ^1^ Institute of Pain Medicine and Special Environmental Medicine Co‐innovation Center of Neuroregeneration Nantong University Jiangsu 226019 China; ^2^ Department of Neurology Mayo Clinic Rochester MN 55905 USA

**Keywords:** c‐MYC, microglia, neuropathic pain, nuclear factor of activated T‐cells (NFAT1), proliferation

## Abstract

Peripheral nerve injury‐induced spinal microglial proliferation plays a pivotal role in neuropathic pain. So far, key intracellular druggable molecules involved in this process are not identified. The nuclear factor of activated T‐cells (NFAT1) is a master regulator of immune cell proliferation. Whether and how NFAT1 modulates spinal microglial proliferation during neuropathic pain remain unknown. Here it is reported that NFAT1 is persistently upregulated in microglia after spinal nerve ligation (SNL), which is regulated by TET2‐mediated DNA demethylation. Global or microglia‐specific deletion of *Nfat1* attenuates SNL‐induced pain and decreases excitatory synaptic transmission of lamina II neurons. Furthermore, deletion of *Nfat1* decreases microglial proliferation and the expression of multiple microglia‐related genes, such as cytokines, transmembrane signaling receptors, and transcription factors. Particularly, SNL increases the binding of NFAT1 with the promoter of *Itgam*, *Tnf*, *Il‐1b*, and *c‐Myc* in the spinal cord. Microglia‐specific overexpression of c‐MYC induces pain hypersensitivity and microglial proliferation. Finally, inhibiting NFAT1 and c‐MYC by intrathecal injection of inhibitor or siRNA alleviates SNL‐induced neuropathic pain. Collectively, NFAT1 is a hub transcription factor that regulates microglial proliferation via c‐MYC and guides the expression of the activated microglia genome. Thus, NFAT1 may be an effective target for treating neuropathic pain.

## Graphical abstract

The study shows that nuclear factor of activated T‐cells (NFAT1) is specifically expressed in spinal microglia and contributes to microglial proliferation via c‐MYC. In addition, NFAT1 controls the expression of multiple genes related to microglial activation and synaptic transmission. Targeting NFAT1/c‐MYC may be effective for the treatment of neuropathic pain.

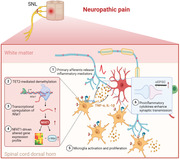

## 1 Introduction

Neuropathic pain resulting from peripheral nerve injury is a debilitating pathological pain condition with a great therapeutic challenge in clinical practice. Nerve injury induces synaptic plasticity in the spinal cord, a driving force for central sensitization and chronic pain.^[^ ^1^ ^]^ Glial cells such as microglia and astrocytes play an important role in regulating synaptic plasticity via releasing inflammatory mediators (such as tumor necrosis factor‐alpha (TNF‐*α)* and interleukin‐1beta (IL‐1*β*)) or growth factors (such as brain‐derived neurotrophic factor (BDNF)) under chronic pain conditions.^[^ ^2^ ^]^ Particularly, intrathecal injection of adenosine triphosphate (ATP)‐activated microglia or selective activation of microglia induces mechanical allodynia, a cardinal symptom of chronic pain.^[^ ^3^ ^]^ Pharmacological or chemogenetic inhibition of spinal microglial function attenuates nerve injury‐induced neuropathic pain,^[^ ^3^, ^4^ ^]^ indicating the pivotal role of spinal microglia in the pathogenesis of chronic pain.

As the resident immune cell of the central nervous system (CNS), microglia transforms into reactive phenotypes in response to injuries and insults.^[^ ^5^ ^]^ Microglia in the spinal cord are strongly activated after peripheral nerve injury, which is largely driven by primary sensory neuron‐derived molecules including ATP, neuregulin‐1, colony‐stimulating factor 1 (CSF1), and chemokines.^[^ ^6^ ^]^ Activated microglia show increased expression of various microglial signaling molecules, including cell‐surface receptors such as chemokine receptors (CX3CR1 and CCR7)^[^ ^6^, ^7^ ^]^ and purinergic receptors (P2X4, P2X7, and P2Y12),^[^ ^3^, ^8^ ^]^ intracellular kinases (p38 and extracellular signal‐regulated kinase (ERK)),^[^ ^6^, ^9^ ^]^ transcription factors (interferon regulatory factor 5 (IRF5) and IRF8),^[^ ^10^ ^]^ and inflammatory mediators (TNF‐*α* and BDNF).^[^ ^11^ ^]^ In addition to activation, the population of microglia is expanded after nerve injury through local microglial proliferation.^[^ ^6^, ^12^ ^]^ Mechanistically, microglial proliferation is partially controlled by receptors such as CSF1R (CSF1 receptor), CX3CR1 (CX3CL1 receptor), and P2Y12 (ATP receptor).^[^ ^6^, ^7^, ^12^ ^]^ Interestingly, DAP12, the adaptor protein of CSF1R is not required for microglial proliferation, although it is necessary for CSF1‐induced upregulation of microglia‐associated genes (including *Itgam*, *Cx3cr1*, *Bdnf*, and *P2rx4*).^[^ ^6a^ ^]^ In addition, IRF8, induced exclusively in spinal microglia after peripheral nerve injury, is required only for microglia‐associated gene expression but not for proliferation.^[^ ^10b^ ^]^ Thus, the intracellular mechanism underlying microglial proliferation in response to nerve injury remains elusive.

The nuclear factor of activated T‐cells (NFATs) is a transcription factor family which consists of at least five members: NFAT1–5. Except for NFAT5, NFAT1–4 act as general intracellular calcium sensors and convey extracellular stimuli into the gene expression machinery.^[^ ^13^ ^]^ Ca^2+^‐activated protein phosphatase calcineurin dephosphorylates NFAT to induce its nucleus translocation and transcription regulation.^[^ ^14^ ^]^ NFAT isoforms are expressed in various immune cells^[^ ^15^ ^]^ and direct the proliferation of different immune and tumor cells.^[^ ^16^ ^]^ It has been reported that NFAT1 and NFAT2 mediate TLR4‐mediated inflammatory responses in primary brain microglia.^[^ ^17^ ^]^ However, whether and how NFATs regulate spinal microglia function and neuropathic pain after peripheral nerve injury have not been investigated.

In this work, we found that NFAT1 (also named NFATc2) is upregulated in spinal microglia after spinal nerve ligation (SNL). Deletion of *Nfat1* attenuated SNL‐induced neuropathic pain and reduced microglial proliferation in the spinal cord. We further identified that c‐MYC is directly regulated by NFAT1, which contributes to microglial proliferation and the development of neuropathic pain.

## 2 Results

### 2.1 SNL Increases NFAT1 Expression in Spinal Microglia

**Figure 1:**
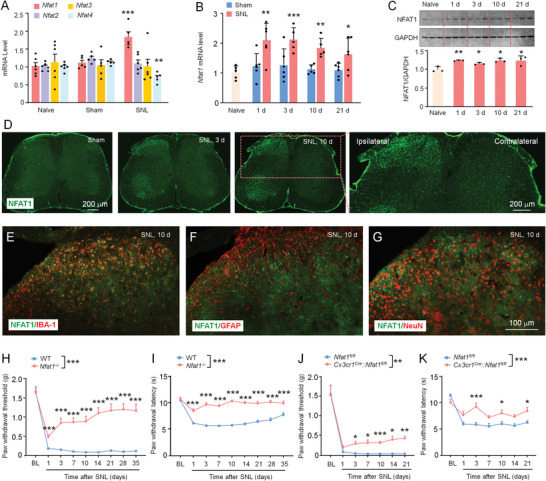
NFAT1 expression is increased in spinal microglia after SNL. A) Real‐time PCR shows the mRNA expression of NFAT1–4 after SNL. ** *P* < 0.01, *** *P* < 0.001, SNL versus Sham; Student's *t*‐test, *n* = 5–6 mice per group. B) The time‐course of *Nfat1* mRNA expression in the ipsilateral dorsal horn in naïve, sham‐ and SNL‐operated mice. * *P* < 0.05, ** *P* < 0.01, *** *P* < 0.001, SNL versus Sham. Student's *t*‐test, *n* = 5–6 mice per group. C) Western blot shows the increased NFAT1 expression in WT mice after SNL from day 1 to day 21. * *P* < 0.05, ** *P* < 0.01, SNL versus Naive. Student's *t*‐test, *n* = 3 mice per group. D) Immunostaining shows NFAT1 expression in the spinal cord of sham and SNL‐operated (day 3 and day 10) mice. E–G) Representative images of double staining of NFAT1 with IBA‐1 (E), GFAP (F), or NeuN (G). NFAT1 is highly colocalized with IBA‐1. H,I) SNL‐induced mechanical allodynia (*F*_(1, 29)_ = 74.30, *P* < 0.0001, H) and heat hyperalgesia (*F*_(1, 29)_ = 214.8, *P* < 0.0001, I) are markedly alleviated in *Nfat1^−/−^* mice compared with WT mice. *** *P* < 0.001, two‐way RM ANOVA followed by Bonferroni's tests, *n* = 15–16 mice per group. J,K) *Nfat1*‐cKO mice exhibit alleviated mechanical allodynia (*F*_(1, 16)_ = 13.64, *P* = 0.002, J) and heat hyperalgesia (*F*_(1, 16)_ = 64.75, *P* < 0.0001, K). * *P* < 0.05, ** *P* < 0.01, *** *P* < 0.001, two‐way ANOVA followed by Bonferroni's multiple comparisons test, *n* = 9 mice per group.

To explore NFATs functions in neuropathic pain, we first used quantitative PCR (qPCR) to compare the expression of *Nfat1–4* mRNA level in the dorsal horn (DH) of naive, sham‐operated, and SNL‐operated mice. We found that SNL markedly increased *Nfat1* (*Nfatc2*) mRNA expression but not the level of *Nfat2 (Nfatc1)*, *Nfat3 (Nfatc4)*, or *Nfat4 (Nfatc3)* mRNA 10 days after SNL (**Figure** **1**A). Furthermore, compared with sham control, *Nfat1* mRNA was increased from 1 day after SNL and maintained for more than 21 days (Figure 1B). Consistently, NFAT1 protein level was also upregulated (Figure 1C), and its immunoreactivity (IR) was increased in the ipsilateral spinal cord compared with contralateral side or sham control (Figure 1D). Moreover, NFAT1 is highly colocalized with microglial marker IBA‐1 (Figure 1E), but not with astrocytic marker GFAP (Figure 1F) or neuronal marker NeuN (Figure 1G), indicating the dominant expression of *Nfat1* in spinal microglia.

To further confirm the cellular distribution of NFAT1 in the spinal cord, a single‐cell RT‐PCR analysis was performed. The contents of GFP‐labeled microglia from *Cx3cr1^Gfp^* mouse, GFP‐labeled astrocytes from *Aldh1l1^Gfp^* mouse, and lamina II neurons from C57Bl/6 mouse were sucked into a glass pipette (Figure S1A, Supporting Information). The amplified products obtained by single‐cell PCR were shown by agarose electrophoresis. As expected, microglia, astrocyte, and neurons expressed their respective markers *Aif1* (the gene encoding IBA‐1), *Gfap*, and *NeuN*. The positive rates of *Nfat1* in microglia, astrocyte, and neuron were 87.5% (7/8), 25% (2/8), and 25% (2/8), respectively (Figure S1B, Supporting Information), confirming the predominant expression of *Nfat1* in spinal microglia.

### 2.2 NFAT1 Is Necessary for the Development and Maintenance of Neuropathic Pain

To assess whether NFAT1 is involved in neuropathic pain, we generated *Nfat1* ^−/−^ mice by CRISP/Cas9 technology (Figure S2A–E, Supporting Information). These *Nfat1* ^−/−^ mice have normal gross anatomy and immune organs (spleen, thymocytes, and lymph node) (Figure S2F–H, Supporting Information), and show comparable acute pain sensation and motor function with WT mice (Figure S3A–D, Supporting Information). However, compared with WT mice, we found that the mechanical allodynia was considerably alleviated in *Nfat1* ^−/−^ mice from 1 day to 35 days after SNL (Figure 1H). Heat hyperalgesia was also persistently attenuated in *Nfat1* ^−/−^ mice (Figure 1I). In addition, formalin‐induced acute inflammatory pain (Figure S3E, Supporting Information) and complete Freund's adjuvant (CFA)‐induced chronic inflammatory pain were reduced in *Nfat1* ^−/−^ mice (Figure S3F,G, Supporting Information).

As NFAF1 is mostly upregulated in spinal microglia after SNL, we next examined the function of microglial NFAT1 in neuropathic pain. To this end, we generated *Nfat^fl/fl^* mice and specifically deleted NFAT1 from microglia using *Nfat1* conditional knockout mice (cKO, *Cx3cr1^Cre^*::*Nfat1^fl/fl^*) (Figure S4A–C, Supporting Information). The cKO mice show normal acute pain thresholds and motor function (Figure S4D–G, Supporting Information). Again, we found that SNL‐induced mechanical allodynia and thermal hyperalgesia were significantly attenuated in *Nfat1* cKO mice (Figure 1J,K). The pain reduction in cKO mice is less than that in *Nfat1* ^−/−^ mice, suggesting that non‐microglial NFAT1 in CNS and/or PNS likely also plays a role in the pathogenesis of neuropathic pain.

### 2.3 NFAT1 Contributes to SNL‐Induced Central Sensitization

**Figure 2:**
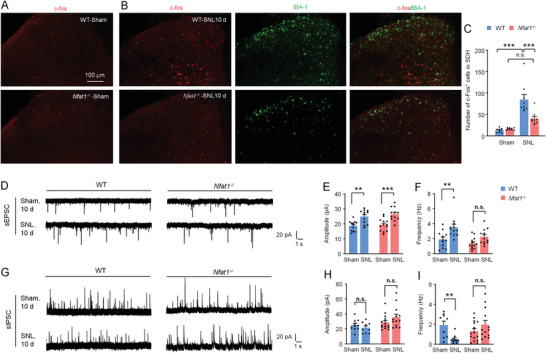
*Nfat1* deletion attenuates neuronal activity and synaptic transmission. A) Immunostaining of c‐Fos in the dorsal horn of naïve WT and *Nfat1* ^−/−^ mice. B) Double staining of c‐Fos and IBA‐1 in WT and *Nfat1* ^−/−^ mice 10 days after SNL. C) Quantification of c‐Fos^+^ cells in the dorsal horn of WT and *Nfat1* ^−/−^ mice in Naïve state or after SNL. *** *P* < 0.001, two‐way ANOVA followed by Bonferroni's test. n.s., no significance. D–F) SNL increases the frequency and amplitude of sEPSC in lamina II neurons of WT mice. Deleting *Nfat1* does not affect the amplitude (D,E) but reduces the frequency of sEPSCs (D,F) compared to WT mice. Frequency: *F*_(1, 45)_ = 7.896, *P* = 0.0073, two‐way ANOVA followed by Bonferroni's test. ** *P* < 0.01, *** *P* < 0.001, SNL versus Naïve. G–I) SNL did not affect the frequency of sIPSC in lamina II neurons of WT or *Nfat1* ^−/−^ mice (G,H). The frequency of sIPSC was reduced in WT mice, not in *Nfat1* ^−/−^ mice after SNL (G,I). ** *P* < 0.01, SNL versus Naïve. n.s., no significance.

Spinal c‐Fos expression was widely used as a functional marker of central spinal sensitization after nerve injury or tissue damage.^[^ ^18^ ^]^ Immunostaining showed that a few c‐Fos^+^ cells are shown in WT‐sham and *Nfat1* ^−/−^‐sham mice (**Figure** **2**A). On SNL Day 10, the number of c‐Fos^+^ cells in dorsal horn neurons was markedly increased in WT mice but was not significantly increased in *Nfat1* ^−/−^ mice (Figure 2B,C). In addition, the intensity of IBA‐1 was lower in *Nfat1* ^−/−^ mice than that in WT mice after SNL (Figure 2B), suggesting that *Nfat1* deletion reduces SNL‐induced spinal neuronal activation and microglial activation.

As enhanced synaptic transmission in dorsal horn neurons has been strongly implicated in neuropathic pain,^[^ ^19^ ^]^ we further performed patch‐clamp recordings in lamina II neurons in which nociceptive information is modulated and conveyed to projection neurons.^[^ ^20^ ^]^ SNL increased the amplitude and frequency of spontaneous excitatory postsynaptic currents (sEPSCs) in WT mice 10 days after SNL. By contrast, SNL increased the amplitude but not the frequency of sEPSCs in *Nfat1* ^−/−^ mice (Figure 2D–F). In addition, SNL decreased the frequency of spontaneous inhibitory postsynaptic currents (sIPSCs) in neurons from WT mice but did not affect the frequency of sIPSCs in neurons from *Nfat1* ^−/−^ mice (Figure 2G–I). These data suggest that SNL‐induced excitatory synaptic transmission is reduced, and SNL‐induced inhibitory synaptic transmission is increased in *Nfat1* ^−/−^ mice.

### 2.4 The Expression of NFAT1 Is Regulated by TET2 Mediated‐DNA Demethylation

**Figure 3:**
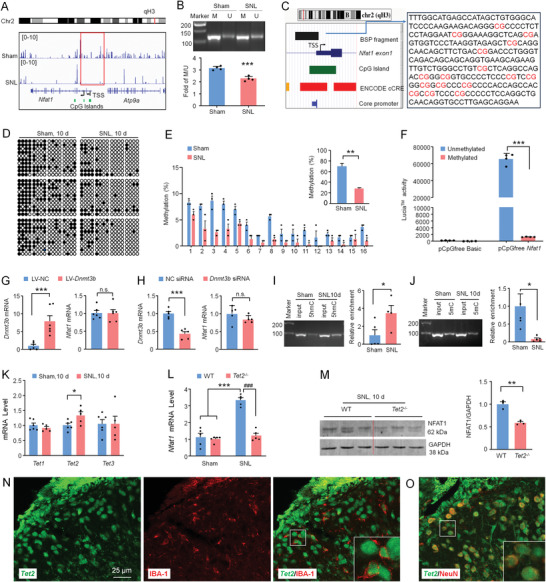
NFAT1 expression is regulated by TET2‐mediated DNA methylation. A) Integrative Genome Browser images were created from MeDIP‐seq data for *Nfat1* gene loci. Black arrows show the transcription start site (TSS) of *Nfat1*. The red box shows a differentially methylated peak upstream of the *Nfat1* gene between sham and SNL. The green blocks represent the location of the CpG islands. B) MSP analysis for *Nfat1* in the dorsal horn collected from Sham and SNL mice. M and U represent methylated and unmethylated bands, respectively. *** *P* < 0.001, SNL versus Sham. C) Left panel: schematic of a CpG island (green block) locus and BSP target fragment (black block) in the promoter of *Nfat1* depicted by the UCSC Genome Browser. Right panel: BSP target fragment. CpG dinucleotides are marked with red font. D) The methylation status of *Nfat1* in the sham‐ and SNL‐operated mice (filled circle, methylated CpG; hollow circle, unmethylated CpG). E) The total methylation of *Nfat1* promoter is decreased after SNL. **P < 0.01, SNL versus Sham, Student's *t*‐test, *n* = 3. F) Methylated luciferase reporter assay shows a decreased luciferase activity of the methylated *Nfat1* promoter compared to the unmethylated one. ***P < 0.001. Student's *t*‐test, *n* = 4. G) Overexpression or H) knockdown of *Dnmt3b* does not affect the expression of *Nfat1*. ***P < 0.001. n.s., no significance. Student's *t*‐test, *n* = 6. Levels of I) 5 hmC and J) 5 mC of *Nfat1* promoter determined by meDIP‐ and hmeDIP‐qPCR, respectively. SNL upregulates 5 hmC level but downregulates 5mC level. *P < 0.05, SNL versus Sham. Student's *t*‐test, *n* = 4–5. K) *Tet1‐3* mRNA expression in the spinal cord of sham and SNL mice. SNL upregulates *Tet2* expression. *P < 0.05, SNL versus Sham. Student's *t*‐test, *n* = 5–6. *Tet2* ^−/−^ mice show L) reduced *Nfat1* mRNA and M) protein after SNL. **P < 0.01, ***P < 0.001, WT‐SNL versus WT‐Sham, ^###^ *P* < 0.001, WT‐SNL versus *Tet2* ^−/−^‐SNL. Student's *t*‐test, *n* = 5 for mRNA, and *n* = 3 for protein. N,O) Combined fluorescent in situ hybridization and immunofluorescence demonstrate *Tet2* mRNA in IBA1‐labeled microglia (N) and NeuN‐labeled neurons (O) in the spinal cord dorsal horn.

DNA methylation, which occurs at CpG sites, especially around transcription start sites (TSS), is an essential epigenetic mechanism controlling gene expression.^[^ ^21^ ^]^ The *Nfat1* promoter contains three candidate CpG islands (CpG island 1, chr2: 168426694–168427600; CpG island 2, chr2: 168415631–168415946; CpG island 3, chr2: 168396250–168397024) (**Figure** **3**A). To check whether *Nfat1* expression is regulated by DNA methylation, we did methylated DNA immunoprecipitation sequencing (MeDIP‐seq), methylation‐specific PCR (MSP), and bisulfite sequencing PCR (BSP). MeDIP‐seq data showed that SNL caused downregulation of the methylation of *Nfat1* gene promoter (Figure 3A). The MSP assay confirmed the decreased methylation degree of *Nfat1* promoter in the spinal cord of SNL mice (Figure 3B). As the CpG island 2 locates near the TSS2 of *Nfat1*, which is the TSS of most *Nfat1* transcripts, the BSP primers were designed to amplify this region (Figure 3C). DNA sequencing was performed on PCR products obtained after the treatment of genomic DNA samples with sodium bisulfite. Consistent with Figure 2A,B, *Nfat1* promoter became demethylated in SNL mice (Figure 3D,E). Furthermore, the in vitro experiment using a luciferase assay showed that luciferase activity for cells transfected with unmethylated pCpG‐free‐*Nfat1*‐promoter‐Lucia vector was more than that of cells transfected with methylated pCpG‐free‐*Nfat1*‐promoter‐Lucia vector (Figure 3F), suggesting that the promoter activity of *Nfat1* is regulated by DNA methylation.

DNA methylation can be regulated by DNA methyltransferases (DNMTs) and ten‐eleven translocation (TET) methylcytosine dioxygenase.^[^ ^22^ ^]^ The expression of DNMT3b, but not DNMT3a or DNMT1 was decreased in the spinal cord after SNL.^[^ ^23^ ^]^ RT‐PCR showed that intraspinal injection of *Dnmt3b* overexpressing lentivirus (LV‐*Dnmt3b*) dramatically increased *Dnmt3b* mRNA level but did not affect *Nfat1* mRNA level (Figure 3G). In addition, intrathecal injection of *Dnmt3b* siRNA significantly reduced *Dnmt3b* mRNA but did not affect *Nfat1* mRNA either (Figure 3H), indicating that NFAT1 expression is not regulated by DNMT3b.

TETs catalyze the conversion of 5‐methylcytosine (5mC) of DNA to 5‐hydroxymethylcytosine (5hmC), thereby altering the epigenetic state of DNA.^[^ ^22^ ^]^ To examine if TETs are involved in the demethylation of *Nfat1* promoter, we examined the level of 5hmC and 5mC on *Nfat1* promoter by hMeDIP and MeDIP‐PCR, respectively. SNL increased 5hmC and decreased 5mC on *Nfat1* promoter (Figure 3I,J), suggesting that TETs may involve increased *Nfat1* expression after SNL. We then checked *Tet1‐3* expression by qPCR. *Tet2* mRNA, but not *Tet1* or *Tet3* mRNA was increased 10 days after SNL (Figure 3K). In *Tet2* ^−/−^ mice, *Nfat1* mRNA level was dramatically reduced compared to WT mice on SNL Day 10 (Figure 3L). NFAT1 protein was also lower in *Tet2* ^−/−^ mice than that in WT mice (Figure 3M). In situ hybridization with immunostaining further showed that *Tet2* was expressed in IBA‐1‐positive microglia (Figure 3N) and NeuN‐positive neurons (Figure 3O). These data indicate that TET2 regulates the NFAT1 expression in spinal microglia after SNL.

### 2.5 *Nfat1* Deletion Reduces Microglial Gene Expression Related to Microglial Proliferation

**Figure 4:**
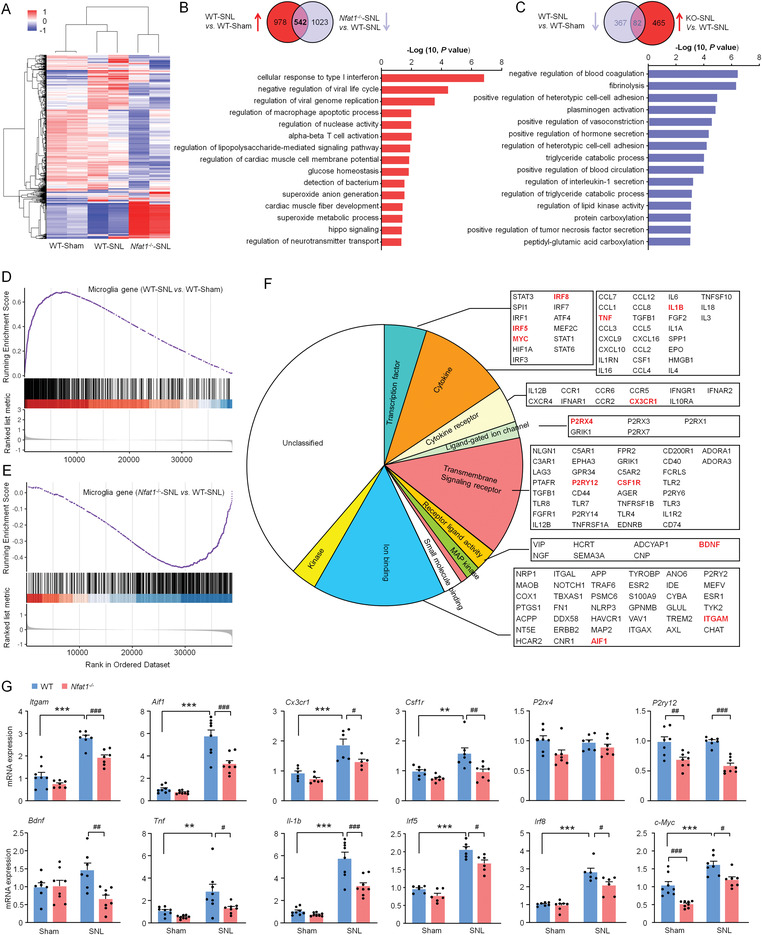
Deletion of *Nfat1* reduces the expression of proliferation‐ and inflammation‐related genes. A) Cluster analysis of the three groups' gene expression profiles: sham‐treated WT (WT‐Sham), SNL‐treated WT (WT‐SNL), and SNL‐treated *Nfat1* ^−/−^ (*Nfat1* ^−/−^‐SNL) mice. B,C) Top panels, Venn diagram showing the intersection of genes that are upregulated by SNL and downregulated by *Nfat1* deletion (B), or downregulated by SNL and upregulated by *Nfat1* deletion (C). Bottom panels: bar charts displaying GO term analysis of genes in the intersections. D,E) GSEA reveals a positive enrichment score for microglia gene set in SNL samples compared to sham samples in WT mice (D), but a negative enrichment score in *Nfat1* ^−/−^‐SNL samples compared to WT‐SNL (E). F) Manual classification of microglia gene function based on literature and gene ontology. G) qRT‐PCR for the microglia‐related gene in WT and *Nfat1^−/−^* mice. ** *P* < 0.01, *** *P* < 0.001, SNL versus Sham. ^#^ *P* < 0.05, ^##^ *P* < 0.01, ^###^ *P* < 0.001, *Nfat1* ^−/−^ versus WT. Two‐way ANOVA followed by Bonferroni's test, *n* = 5–8 mice per group.

To explore the mechanism of microglial NFAT1 underlying neuropathic pain, we performed a microarray to compare gene expression in the spinal cord of *Nfat1* ^−/−^ and WT mice after SNL (**Figure** **4**A). Compared to WT‐sham mice, 1520 genes were upregulated (>1.5‐fold) in the DH of WT‐SNL mice, while among them, 542 of these genes were downregulated in *Nfat1* ^−/−^‐SNL mice (Figure 4B). Conversely, in 449 genes downregulated in WT‐SNL mice, 82 of them were upregulated in *Nfat1* ^−/−^‐SNL mice (Figure 4C). Gene ontology (GO) analysis revealed that the downregulated genes in response to *Nfat1* deletion after SNL are mainly associated with the immune response‐related biological processes, including cellular response to type I interferon, regulation of macrophage apoptotic process, and regulation of lipopolysaccharide‐mediated signaling pathway (Figure 4B). However, the upregulated genes in response to *Nfat1* deletion are associated with negative regulation of blood coagulation, fibrinolysis, positive regulation of heterotypic cell–cell adhesion, and plasminogen activation (Figure 4C). Thus, these results suggest that NFAT1 is pivotal in immune regulation and tissue repair after SNL.

To further analyze whether NFAT1 regulates the expression of microglia‐specific genes, we performed gene set enrichment analysis (GSEA) comparing WT‐SNL versus WT‐Sham (Figure 4D) and *Nfat1* ^−/−^‐SNL versus WT‐SNL (Figure 4E). We used the custom gene set of “microglia genes” which contains 254 genes specifically expressed in microglia. GSEA plots showed significant enrichment of the “microglia genes” for the upregulated genes after SNL (Figure 4D). By contrast, the “microglia genes” were substantially downregulated in *Nfat1* ^−/−^‐SNL mice compared with WT‐SNL mice (Figure 4E). In these downregulated genes from *Nfat1* ^−/−^‐SNL mice, GO analysis showed enrichment for ion binding and transmembrane signaling receptor genes, followed by genes related to cytokine, cytokine receptor, and transcription factors (Figure 4F). Among those, we highlighted microglial markers (such as *Itgam*, the gene encoding CD11b, and *Aif1*, the gene encoding IBA‐1) and molecules involving microglial proliferation and activation, including cytokines and receptors (such as *Tnf*, *Il1b*, and *Csf1r*), chemokine receptors (such as *Cx3cr1*), purinergic receptors (such as *P2rx4* and *P2ry12*), and transcriptional factors (such as *Irf5*, *Irf8*, and *Myc*) (Figure 4F). We used qPCR to validate the mRNA levels of the genes mentioned above in the spinal cord of WT mice and *Nfat1* ^−/−^ mice. Consistently, most of these genes were upregulated after SNL in WT mice and downregulated in *Nfat1* ^−/−^‐SNL mice (Figure 4G). These data suggest that NFAT1 regulates the expression of multiple genes related to microglial activation and proliferation.

### 2.6 *Nfat1* Deletion Reduces SNL‐Induced Microglial Proliferation

**Figure 5:**
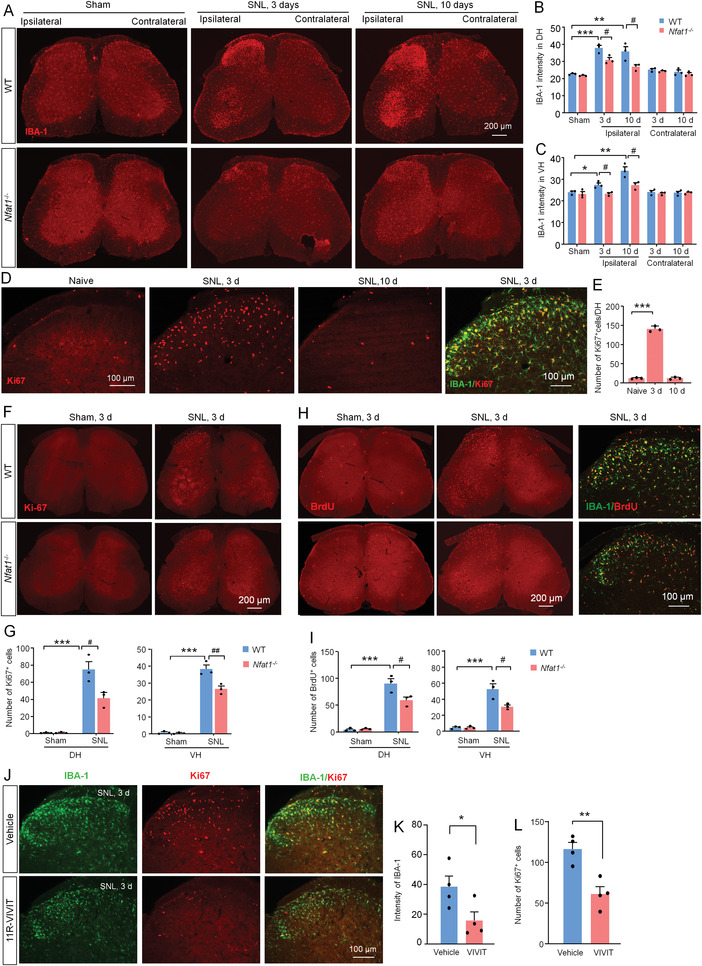
NFAT1 contributes to SNL‐induced microglial activation and proliferation in the spinal cord. A) The immunofluorescence staining of IBA‐1 in the spinal cord from naïve or SNL‐operated WT and *Nfat1^−/−^* mice. B,C) Immunofluorescence intensity analysis shows that the immunoreactivity of IBA‐1 in the ipsilateral dorsal horn (DH); B) and ventral horn (VH; C) was lower in *Nfat1^−/−^* mice than that in WT mice. * *P* < 0.05, **P < 0.01, ***P < 0.001, WT‐SNL versus WT‐Naïve; ^#^ *P* < 0.05, *Nfat1* ^−/−^‐SNL versus WT‐SNL. Student's *t*‐test, *n* = 3. D) The expression of Ki67 in the spinal cord and double staining of Ki67 and IBA‐1. E) The number of Ki67^+^ cells is dramatically increased 3 days after SNL. ***P < 0.001, one‐way ANOVA followed by Bonferroni's multiple comparisons test, *n* = 3. F) Ki67 staining in the spinal cord from WT and *Nfat1* ^−/−^ mice 3 days after SNL. G) Quantification shows that the number of Ki67^+^ cells in the DH and VH is lower in *Nfat1* ^−/−^ mice than that in WT mice. ***P < 0.001, WT‐SNL versus WT‐Naïve. ^#^ *P* < 0.05, *Nfat1* ^−/−^‐SNL versus WT‐SNL, two‐way ANOVA followed by Bonferroni's test, *n* = 3. H) BrdU distribution in WT mice and *Nfat1* ^−/−^ mice and double staining of BrdU and IBA‐1. I) Quantification shows that the number of BrdU^+^ cells is reduced in the DH and VH of *Nfat1* ^−/−^ mice compared to WT mice. ***P < 0.001, SNL versus Naïve, ^#^ *P* < 0.05, *Nfat1* ^−/−^ versus WT, two‐way ANOVA followed by Bonferroni's test, *n* = 3. J) Double immunofluorescence of IBA‐1 and Ki67 in the spinal cord from SNL mice treated with 11R‐VIVIT or vehicle. The intensity of K) IBA‐1^+^ and L) the number of Ki67^+^ cells are decreased by 11R‐VIVIT. * *P* < 0.05, ** *P* < 0.01, Student's *t*‐test, *n* = 4 mice per group.

We next examined the function of NFAT1 in spinal microglia after peripheral nerve injury. In WT mice, IBA‐1 immunoreactivity was dramatically increased in the ipsilateral DH and VH at Day 3 and Day 10 after SNL (**Figure** **5**A). By contrast, the IBA‐1 expression in *Nfat1* ^−/−^ mice was much lower than that in WT mice (Figure 5B,C). Furthermore, p38 MAPK, which is specifically expressed in spinal microglia of mice after SNL (Figure S5A, Supporting Information),^[^ ^9^ ^]^ was also remarkably reduced in *Nfat1* ^−/−^ mice (Figure S5B,C, Supporting Information).

The reduced IBA‐1 intensity after NFAT1 deletion suggests its function in microglial proliferation after SNL. To test this idea, we performed the staining of Ki67, a marker for cell proliferation. Indeed, Ki67 was dramatically increased on Day 3 after SNL and was largely colocalized with IBA‐1 in WT mice (Figure 5D,E). When we examined Ki67 expression in *Nfat1* ^−/−^ mice after SNL, we found that the numbers of Ki67^+^ cells in the DH and VH of *Nfat1* ^−/−^ mice were significantly reduced (Figure 5F,G). We further confirmed the role of NFAT1 in microglial proliferation using BrdU staining. Consistently, the number of BrdU^+^ cells was lower in *Nfat1* ^−/−^ mice than in WT mice after SNL, and BrdU was also predominantly colocalized with IBA‐1 (Figure 5H,I). To further confirm the role of NFAT1 in SNL‐induced microglial proliferation, we used an inhibitory peptide 11R‐VIVIT, which interacts with NFAT at its calcineurin binding site and inhibits its activation.^[^ ^24^ ^]^ Twenty‐four hours after intrathecal (i.t.) injection of 11R‐VIVIT (1 nmol) on SNL Day 3, the intensity of IBA‐1‐IR and the number of Ki67^+^ cells were significantly reduced (Figure 5J–L). Together, these data indicate that NFAT1 plays an important role in SNL‐induced spinal microglial proliferation.

### 2.7 NFAT1 Directly Regulates Microglial Gene Expression In Vitro and In Vivo

**Figure 6:**
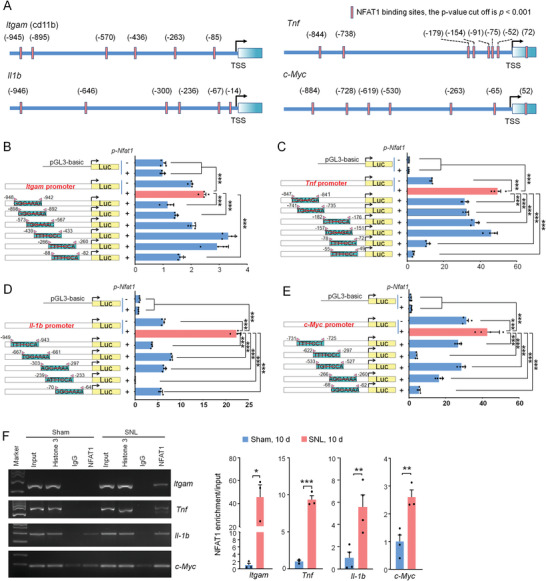
NFAT1 directly regulates the transcription of *Itgam*, *Tnf*, *Il‐1b*, and *c‐Myc*. A) The transcription binding motif of NFAT1 in *Itgam*, *Tnf*, *Il‐1b*, and c‐*Myc* was predicted by the JASPAR CORE vertebrate database. B–D) Diagrams at the left of each panel illustrate the number and distribution of NFAT1 binding sites in the promoter regions of *Itgam* (B), *Tnf* (C), *Il‐1b* (D), and *c‐Myc* (E). The luciferase report assay (the right part of each panel) shows that overexpression of NFAT1 promotes the activity of the promoters of *Itgam*, *Tnf*, *Il‐1b*, and *c‐Myc*. Mutations of some of these predicted NFAT1 binding sites reduce NFAT1‐stimulated luciferase activity mediated by their corresponding genes promoter. *** *P* < 0.001, comparison between WT promoter with *Nfat1* overexpression groups (red bar) and other groups (blue bar). One‐way ANOVA followed by Bonferroni's tests, *n* = 4 per group. F) ChIP‐PCR shows that the binding of NFAT1 with *Itgam*, *Tnf*, *Il‐1b*, and *c‐Myc* is increased after SNL. * *P* < 0.05, ** *P* < 0.01, *** *P* < 0.001. Student's *t*‐test, *n* = 3–4 per group.

To explore the molecular mechanism of NFAT1 in microglial function, we further analyzed the gene expression regulated by NFAT1. Using transcription factor binding motif enrichment analysis, we found that the NFAT1‐binding motif is enriched within the promoter region of gene downregulated after NFAT1 deletion (Figure S6A, Supporting Information). We further analyzed the sequence of the promoters of these genes by JASPAR CORE and observed that the promoters of *Itgam*, *Tnf*, *Il1b*, and *c‐Myc* have more than 5 binding sites of NFAT1 (**Figure** **6**A), and many others have 1–4 binding sites (Figure S6B, Supporting Information).

To examine the binding site(s) of NFAT1 on the promoter essential for the gene expression, we made site‐directed mutagenesis of putative NFAT1 consensus binding sites (BS) for the promoter using luciferase activity assay. We focus on four genes (*Itgam*, *Tnf*, *Il1b*, and *c‐Myc*) that have more than five binding sites of NFAT1. The mutation of BS1, BS2, or BS6 on *Itgam* promoter significantly reduced the transactivation effect of NFAT1 (Figure 6B). Mutation of BS1, BS2, BS3, BS5, or BS6 on *Tnf* promoter, and mutation of BS1‐5 of *Il1b* promoter markedly reduced the transactivation effect of NFAT1 (Figure 6C,D). For *c‐Myc* gene, mutation of any of the five binding sites of NFAT1 with *c‐Myc* promoter reduced the luciferase activity (Figure 6E). These results indicate that NFAT1 directly regulates the transcription of *Itgam*, *Tnf*, *Il1b*, and *c‐Myc*.

To understand the gene regulation by NFAT1 in vivo, we then used chromatin immunoprecipitation‐PCR (ChIP‐PCR) to compare the binding of NFAT1 with these genes in the spinal cord of sham‐ and SNL‐operated mice. The data showed that the promoters of *Itgam*, *Tnf*, *Il‐1b*, and *c‐Myc* have higher enrichment of NFAT1 occupancy in the spinal cord of SNL‐operated mice compared with sham‐operated mice (Figure 6F). Thus, the binding of NFAT1 with the promoter of these genes in the spinal cord is enhanced after SNL.

### 2.8 c‐MYC Contributes to Microglial Proliferation and Neuropathic Pain

**Figure 7:**
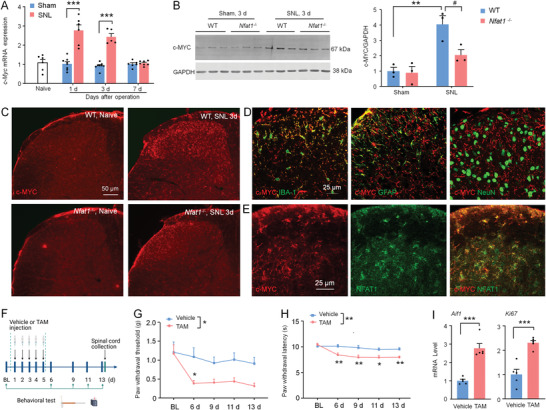
c‐MYC is involved in SNL‐induced microglial proliferation and neuropathic pain. A) The *c‐Myc* mRNA expression in naïve, sham‐, and SNL‐operated mice. *** *P* < 0.001, versus sham. Student's *t*‐test. *n* = 5‐8 mice per group. B) Western blot showing c‐MYC protein level in the spinal cord. ** *P* < 0.01, SNL versus Sham. ^##^ *P* < 0.05, *Nfat1* ^−/−^ versus WT, Student's *t*‐test. *n* = 3 mice per group. C) Fluorescence staining showing c‐MYC expression in the spinal cord of WT and *Nfat1* ^−/−^ mice after SNL. D) Representative images of double staining of c‐MYC with IBA‐1, GFAP, and NeuN. E) Double staining of c‐MYC and NFAT1. F) Experimental timeline of procedures, treatment administration, pain behavioral testing, and tissue collection. G,H) Tamoxifen (TAM) induces mechanical allodynia (G) and thermal hyperalgesia (H) in H11‐LSL‐Myc::Cx3cr1^CreERT2^ mice. For mechanical allodynia, *F*_(1, 22)_ = 7.614, P = 0.0114; For heat hyperalgesia, *F*_(1, 22)_ = 13.61, *P* = 0.0013; *** *P* < 0.001, TAM versus vehicle. Two‐way ANOVA followed by Bonferroni's tests. *n* = 12 mice per group. I) TAM treatment increases the levels of *Aif1* and *Ki67* in the spinal cord of H11‐LSL‐Myc::*Cx3cr1* ^CreERT2^ mice. *** *P* < 0.001, TAM versus vehicle. Student's *t*‐test.

It is well known that TNF‐*α* and IL‐1*β* play an important role in chronic pain,^[^ ^20^, ^25^ ^]^ but the role of c‐MYC in neuropathic pain has not been reported. Thus, we investigated the function of c‐MYC in neuropathic pain. The qRT‐PCR results showed that *c‐Myc* mRNA was significantly increased at Day 1 and Day 3, but not at Day 7 after SNL compared with sham control (**Figure** **7**A). At the protein level, SNL‐induced upregulation of c‐MYC protein was abolished in *Nfat1* ^−/−^ mice (Figure 7B). Consistently, immunostaining showed that c‐MYC was markedly increased 3 days after SNL in WT mice, and the upregulation was diminished in *Nfat1* ^−/−^ mice (Figure 7C). Furthermore, c‐MYC was mostly colocalized with microglial marker IBA‐1 (Figure 7D) and with NFAT1 (Figure 7E). Thus, the results suggest that c‐MYC is likely downstream of NFAT1 in spinal microglia after peripheral nerve injury.

To assess the function of microglial c‐MYC in pain behaviors, we conditionally overexpressed c‐MYC in microglia using H11‐LSL‐Myc::*Cx3cr1* ^CreERT2^ mice which express *c‐Myc* following excision of the transcriptional stop cassette by *Cre* after tamoxifen (TAM) treatment (Figure S7, Supporting Information). We injected TAM or vehicle (castor oil, intraperitoneally (i.p.)) for 5 consecutive days and then performed behavioral tests (Figure 7F). Interestingly, we found that TAM‐treated mice displayed thermal hyperalgesia and mechanical allodynia compared to the vehicle group (Figure 7G,H). In addition, *Aif1* and *Ki67* were also significantly upregulated in TAM‐treated mice compared with vehicles (Figure 7I). These results indicate that selective overexpression of c‐MYC in microglia is sufficient to induce pain phenotypes and microglial proliferation.

### 2.9 Targeting NFAT1/c‐MYC Attenuates Neuropathic Pain

**Figure 8:**
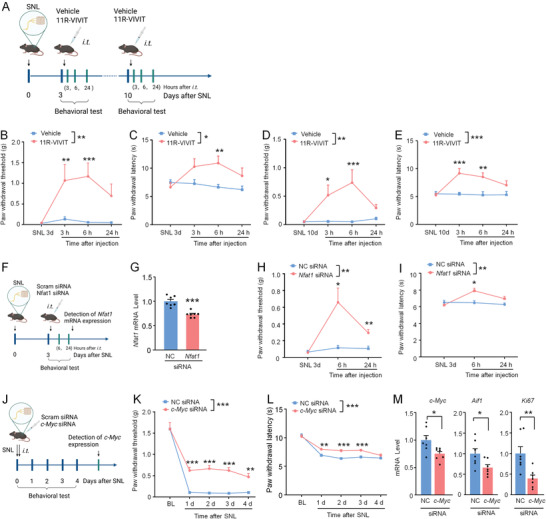
NFAT1/c‐MYC contributes to the pathogenesis of neuropathic pain. A) Experimental timeline of SNL, drug administration, and pain behavioral testing. B,C) The 11R‐VIVIT on SNL Day 3 attenuates mechanical allodynia (Treatment, *F*_(1, 13)_ = 16.14, *P* = 0.0015, B) and heat hyperalgesia (*F*_(1, 13)_ = 5.244, *P* = 0.0394, C). * *P* < 0.05, ** *P* < 0.01. Two‐way ANOVA followed by Bonferroni's tests, *n* = 7–8 mice per group. D,E) The 11R‐VIVIT on SNL Day 10 attenuates mechanical allodynia (Treatment, *F*_(1, 14)_ = 10.62, *P* = 0.0057, D) and heat hyperalgesia (*F*_(1, 14)_ = 19.28, *P* = 0.0006, E). * *P* < 0.05, ** *P* < 0.01, *** *P* < 0.001. Two‐way ANOVA followed by Bonferroni's tests, *n* = 8 mice per group. F) Experimental timeline of SNL, siRNA administration, pain behavioral testing, and the spinal cord collection. G) qRT‐PCR showing the knockdown efficiency of *Nfat1* siRNA. *** *P* < 0.001, *n* = 7 mice per group. H,I) *Nfat1* siRNA alleviates SNL‐induced mechanical allodynia (*F*_(1, 14)_ = 12.48, *P* = 0.0033, H) and heat hyperalgesia (*F*_(1, 14)_ = 10.98, *P* = 0.0051, I) after SNL. * *P* < 0.05, ** *P* < 0.01, two‐way RM ANOVA followed by Bonferroni's tests, *n* = 8 mice per group. J) Experimental timeline of SNL, siRNA administration, pain behavioral testing, and the spinal cord collection. K,L) *c‐Myc* siRNA alleviates SNL‐induced mechanical allodynia (K) and heat hyperalgesia (L) after SNL. For mechanical allodynia: *F*_(1, 30)_ = 45.11, *P* < 0.0001. For heat hyperalgesia: *F*_(1, 30)_ = 36.66, *P* < 0.0001; *c‐Myc* siRNA versus NC siRNA, two‐way RM ANOVA followed by Bonferroni's tests, * *P* < 0.05, ** *P* < 0.01, *** *P* < 0.001, *n* = 7–8 mice per group. M) qRT‐PCR showing the knockdown efficiency of *c‐Myc* siRNA. Knockdown of *c‐Myc* also decreases the expression of *Aif1* and *Ki67*. * *P* < 0.05, ** *P* < 0.01, Student's *t*‐test, *n* = 7 mice per group.

Our results collectively suggest the critical role of microglial NFAT1/c‐MYC in neuropathic pain after peripheral nerve injury. We next examined whether inhibition of NFAT1 or c‐MYC attenuates neuropathic pain using pharmacological and genetic manipulations. First, we i.t. injected NFAT1 inhibitor 11R‐VIVIT (1 nmol) after SNL and examined pain behaviors (**Figure** **8**A). Indeed, 11R‐VIVIT remarkably attenuated SNL‐induced mechanical allodynia and heat hyperalgesia 3 and 6 h after injection when injected either on Day 3 or Day 10 after SNL (Figure 8B–E). Second, we knockdown NFAT expression via *Nfat1* siRNA and examined its behavioral effects (Figure 8F). *Nfat1* siRNA treatment reduced *Nfat1* mRNA level by 34.4 ± 3.9% in the spinal cord 24 h after injection (Figure 8G). Compared with NC siRNA, i.t. injection of *Nfat1* siRNA attenuated SNL‐induced mechanical allodynia (Figure 8H) and heat hyperalgesia (Figure 8I). Therefore, both pharmacological inhibition and genetic knockdown of NFAT1 reduced pain sensitization after peripheral nerve injury.

We have shown that c‐MYC is the downstream signaling after NFAT1 in microglia. We speculated that c‐MYC inhibition could also effectively relieve SNL‐induced neuropathic pain. To this end, *c‐Myc* siRNA or NC siRNA, modified with 5′‐cholesteryl and 2′‐O‐methylribonucleotide to enhance the stability and delivery efficiency, was i.t. injected right after SNL (Figure 8J). Indeed, the *c‐Myc* siRNA dramatically attenuated SNL‐induced mechanical allodynia on Day 1–3 after injection (Figure 8K) or SNL‐induced heat hyperalgesia on Day 1 after SNL (Figure 8L). In addition, *c‐Myc* siRNA reduced the mRNA level of *c‐Myc*, *Aif1*, and *Ki67* (Figure 8M). Together, our results demonstrate that NFAT1 and c‐MYC could be targeted for the treatment of neuropathic pain after peripheral nerve injury.

## 3 Discussion

Although numerous studies have reported nerve injury‐induced microglial activation,^[^ ^6^, ^8^, ^9^, ^10^ ^]^ the intracellular mechanisms underlying microglial proliferation remain largely unknown. The current study demonstrated that NFAT1 is a critical transcription factor in regulating the expression of microglia‐related genes and further contributes to microglial proliferation after nerve injury. Among them, c‐MYC is an important downstream signal of NFAT1, which is highly expressed in spinal microglia and critical for microglial proliferation. Genetic deletion or pharmacological inhibition of NFAT1/c‐MYC in microglia attenuated SNL‐induced neuropathic pain. Thus, NFAT1/c‐MYC represents potential therapeutic targets for the management of neuropathic pain.

### 3.1 NFAT1 Is Upregulated in Spinal Microglia by SNL and Contributes to Neuropathic Pain

NFAT was originally found in nuclear extracts from activated T cells and was expressed in various cells.^[^ ^14^, ^15^ ^]^ In the nervous system, NFAT1–4 are expressed in neurons and glial cells. Neuronal NFAT isoforms (such as NFAT3 and NFAT1) are involved in regulating neuronal survival, apoptosis, and axonal growth during development or injury.^[^ ^26^ ^]^ NFAT1 is expressed in primary microglia and contributes to proinflammatory responses.^[^ ^15^, ^17^ ^]^ However, a recent study reported that NFAT1 is expressed in spinal neurons after paclitaxel injection in rats.^[^ ^27^ ^]^ We found that NFAT1 was largely expressed in spinal microglia and increased after SNL in mice. Thus, there might be context‐ or species‐specific cellular distribution of NFAT1.

Recent studies indicate that chemogenetic inhibition of spinal microglial function attenuates nerve injury‐induced neuropathic pain.^[^ ^4a,e^ ^]^ Consistently, our results showed that global deletion of NFAT1 reduced microglial activation and SNL‐induced pain sensitization. In addition, formalin‐induced spontaneous pain and CFA‐induced pain hypersensitivity were alleviated in *Nfat1* ^−/−^ mice, indicating that NFAT1 also plays a role in acute/chronic inflammatory pain. Our data further showed that specific deletion of NFAT1 in microglia alleviated SNL‐induced pain hypersensitivity. However, the NFAT1 cKO mice show less analgesic magnitude than global KO mice. Thus, we believe that NFAT1 in spinal neurons or in the periphery may also contribute to the pathogenesis of neuropathic pain.

DNA methylation plays an important role in regulating gene expression, which involves transferring a methyl group to cysteine residues at CpG sites in the promoter regions of genes. NFAT1 promoter has a large amount of CpG, and SNL caused marked demethylation of NFAT1 promoter. DNMT3b, but not DNMT3a or DNMT1, is downregulated in the spinal cord after SNL and contributes to the demethylation of *Cxcr3* promoter and *Gpr151* promoter.^[^ ^23^, ^28^ ^]^ However, overexpression or inhibition of DNMT3b in the spinal cord did not change *Nfat1* level. By contrast, SNL increased 5hmC and decreased 5mC in the spinal cord, and NFAT1 expression was reduced in *Tet2* ^−/−^ mice after SNL, indicating the regulation of NFAT1 expression by TET2‐mediated hydroxymethylation. In line with our study, TET2 is increased in primary microglia from mouse, rat, or human after LPS incubation and plays a proinflammatory role.^[^ ^29^ ^]^ However, TET2 actively represses IL‐6 transcription during inflammation resolution in dendritic cells and macrophages,^[^ ^30^ ^]^ suggesting that the action of TET2 is context‐specific. Additionally, the proliferation rate of microglia lacking TET2 is decreased upon LPS treatment,^[^ ^29^ ^]^ which supports the role of NFAT1 in microglial proliferation. TET2 is also expressed in neurons in the brain and involved in regulating cell survival,^[^ ^31^ ^]^ thus the role of TET2 in spinal neurons remains to be investigated in the future.

### 3.2 NFAT1 Is Necessary for SNL‐induced Spinal Microglial Activation and Proliferation

NFATs are activated by intracellular Ca^2+^ signals in concert with calcineurin.^[^ ^32^ ^]^ Microglia have low spontaneous calcium activity under normal conditions, while inflammation and injury increase microglial calcium signaling.^[^ ^33^ ^]^ A wide variety of extracellular molecules, purine molecules (ATP, ADP, and UDP), and immune mediators (CX3CL1, CCL5, C3a, C5a, and TNF‐*α*) have been identified to induce calcium transients in microglia.^[^ ^33e^ ^]^ Previous reports have shown that nerve injury increases the expression of immune mediators (such as CX3CL1, CSF1, and TNF‐*α*) and purinergic receptors (such as P2X4 and P2Y12) in the spinal cord.^[^ ^6^, ^34^ ^]^ Thus, NFAT1 may respond to the immune‐ or purinergic receptor‐mediated Ca^2+^ transients to translocate from the cytoplasm to the nucleus.

As a transcription factor, NFAT1 binds to a GGAAA (TTTCC) consensus sequence, which is found in the promoter/enhancer regions of many immune response genes (including IL‐2, IL‐4, and IFN‐*γ*).^[^ ^35^ ^]^ Our data demonstrated that *Nfat1* deletion affected the expression of various genes that are mainly associated with the immune response‐related biological processes. Furthermore, GSEA shows the important role of NFAT1 in controlling the expression of microglia‐related genes: 1) *Csf1r*, *Cx3cr1*, and *P2ry12* are important modulators for microglial proliferation;^[^ ^6^, ^8^, ^12^ ^]^ 2) *Itgam*, *Aif1*, *Tlr2*, *Il‐1b*, *Irf5*, *and Irf8* are associated with a reactive state of microglia.^[^ ^10^ ^]^ Consistently, SNL‐induced microglial activation (IBA‐1 and p‐p38 upregulation) and proliferation (IBA1^+^Ki67^+^ and IBA^+^BrdU^+^ cells) was reduced in *Nfat1* ^−/−^ mice. In addition, NFAT1 inhibitor 11R‐VIVIT administration reduced Ki67^+^/IBA‐1^+^ cells and IBA‐1 expression after SNL. These data collectively indicate an essential role of NFAT1 in spinal microglial proliferation and activation after nerve injury.

### 3.3 NFAT1 Shapes Microglia Activation‐Specific Gene Expression Profile and Upregulates Transcription of the Potent Proinflammatory Gene *Tnf* and *Il‐1b* after SNL

We found that the deletion of *Nfat1* changed the expression of a variety of genes, including *Itgam*, *Tnf*, *Il‐1b*, and *c‐Myc*, which NFAT1 directly regulates. *Itgam*, which encodes CD11b (OX42), is a member of the integrin family and is highly expressed on monocytes/macrophages, dendritic cells, and microglia.^[^ ^36^ ^]^ The activated microglia is associated with increased expression of CD11b.^[^ ^9^, ^10^ ^]^ Our data showed that SNL increased the binding of NFAT1 with *Itgam* promoter in the spinal cord, suggesting that NFAT1 contributes to microglial morphological changes via increasing CD11b expression.

It was well‐demonstrated that TNF‐*α* and IL‐1*β* are released from glial cells and exaggerate pain hypersensitivity^[^ ^4^, ^11^, ^25^, ^37^ ^]^ but the transcriptional regulation mechanism of TNF‐*α* and IL‐1*β* expression is less investigated. We found the increased binding of NFAT1 with the promoter of TNF‐*α* and IL‐1*β* after SNL and identified the critical binding sites. TNFR1 (TNF‐*α* receptor) and IL‐1R (IL‐1*β* receptor) are expressed on spinal dorsal horn neurons and primary afferents.^[^ ^25^, ^38^ ^]^ Perfusion of TNF‐*α* very rapidly increases the frequency of sEPSCs and potentiates NMDA‐induced currents in neurons of lamina II.^[^ ^20^, ^39^ ^]^ Microglia, via releasing TNF‐*α*, mediates caspase 6‐induced increase of the sEPSCs and evoked EPSCs.^[^ ^11a^ ^]^ In addition, IL‐1*β* enhances the function of presynaptic NMDARs, leading to increased glutamate release and excitatory synaptic transmission.^[^ ^20^, ^40^ ^]^ IL‐1*β* also reduces the frequency and amplitude of sIPSCs.^[^ ^20^ ^]^ *Nfat1* deficiency reduced the increase of the frequency of sEPSCs, and blocked the decrease of the frequency of sIPSCs of lamina II neurons, which coincides with the decreased TNF‐*α* and IL‐1*β* in the spinal cord of these mice. Besides the neuronal expression of TNFR1 and IL‐1R, they are expressed in astrocytes and microglia and contribute to glial activation.^[^ ^25^, ^37^, ^41^ ^]^ TNF‐*α* and IL‐1*β* induce glia‐mediated synaptic long‐term potentiation in the spinal cord lamina I neurons and thermal hyperalgesia.^[^ ^37^ ^]^ Thus, NFAT1‐mediated TNF‐*α* and IL‐1*β* upregulation facilitates synaptic transmission and glial activation/proliferation after peripheral nerve injury.

### 3.4 NFAT1 Increases *c‐Myc* Expression to Induce Microglial Proliferation and the Development of Neuropathic Pain

NFAT1 deletion reduced the expression of several transcription factors, including IRFs, STATs, and MYC. Previous studies showed that IRF5 and IRF8 are exclusively expressed in spinal microglia and mediate nerve injury‐induced microglial gene expression.^[^ ^10^ ^]^ c‐MYC has been implicated in the regulation of a variety of biological processes, including growth, differentiation, apoptosis, DNA repair, and protein synthesis.^[^ ^42^ ^]^ Evidence showed that NFAT2 binds to the promoter of *c‐Myc* to accelerate pancreatic cancer cell proliferation.^[^ ^43^ ^]^ Here, we identified five important binding sites of NFAT1 with *c‐Myc* promoter, and NFAT1 was colocalized with c‐MYC in spinal microglia. Additionally, overexpression of *c‐Myc* is sufficient to increase microglial proliferation and induce pain hypersensitivity, while knockdown of *c‐Myc* by siRNA attenuates SNL‐induced chronic pain and reduces Ki67 expression. In line with the present study, inhibition of microglial proliferation reduces spinal nerve transection‐induced pain hypersensitivity.^[^ ^12a^ ^]^ Given that the *c‐Myc* mRNA was only increased in the early 3 days after SNL, c‐MYC may play an important role in the induction but not the maintenance of neuropathic pain.

Our data further showed that intrathecal injection of *Nfat1* siRNA or NFAT1 inhibitor attenuated SNL‐induced neuropathic pain on SNL day 3 and Day 10, indicating the role of NFAT1 in the maintenance of neuropathic pain. Consistently, 11R‐VIVIT or the calcineurin inhibitor FK‐506 attenuates the development of nerve injury‐induced tactile allodynia in rats.^[^ ^44^ ^]^ It is worth noting that although NFAT1 plays a critical role in the spinal cord, we cannot exclude the contribution of peripheral NFAT1 in neuropathic pain, as i.t. injection of siRNA or inhibitor also affects NFAT1 in the DRGs.^[^ ^45^ ^]^ Furthermore, other genes such as BDNF and CCR2 may also be regulated by NFAT1 and contribute to synaptic transmission.^[^ ^14^, ^46^ ^]^ In addition, accumulating evidence supports that several microglia‐expressing genes such as P2X4, BDNF, and p38 mediate pain in male mice.^[^ ^2^, ^47^ ^]^ Whether NFAT1 is involved in the sex difference of chronic pain remains to be determined.

**Figure 9:**
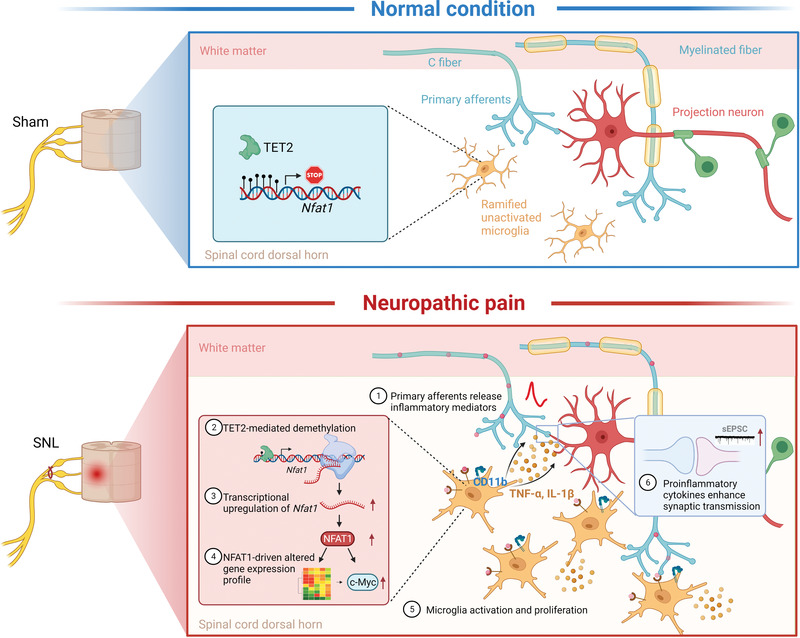
Schematic diagram displaying the role and mechanism of NFAT1 in neuropathic pain. The upper panel shows a low *Nfat1* level in spinal microglia with a hypermethylated *Nfat1* promoter region under normal conditions. The lower panel illustrates the mechanism of NFAT1 in microglial proliferation and central sensitization. First, peripheral nerve injury causes the release of inflammatory mediators from primary afferents to trigger microglial activation. Next, undefined intracellular signaling pathways modulate TET2 upregulation to promote demethylation of the *Nfat1* promoter resulting in the upregulation of NFAT1. Then, NFAT1 causes the reprogramming of microglia gene expression and promotes the proliferation of microglia by upregulating c‐MYC. Finally, the activated microglia release more cytokines such as TNF‐*α* and IL‐1*β* and induce a inflammatory microenvironment that enhances excitatory synaptic transmission, eventually leading to central sensitization and neuropathic pain. The schematic summarizing the results was created using BioRender.

In conclusion, we revealed that NFAT1 is an important transcription factor in regulating microglial proliferation and pathogenesis of neuropathic pain. We propose that microglial NFAT1 may participate in chronic pain in several folds (**Figure** **9**): After peripheral nerve injury, microglial NFAT1 is upregulated due to the TET2‐mediated demethylation of the *Nfat1* promoter, which increases the expression of CD11b to contribute to the microglial activation and the expression of proinflammatory cytokines (such as TNF‐*α* and IL‐1*β*) to contribute to synaptic transmission. In addition, NFAT1 increased the expression of c‐MYC to increase microglial proliferation which is critical for pain hypersensitivity after peripheral nerve injury. Thus, targeting NFAT1 may provide an effective approach for the treatment of neuropathic pain.

## 4 Experimental Section

### Animals and Surgery

Healthy male ICR mice and C57BL/6 mice (20–25 g) were purchased from the Experimental Animal Center of Nantong University. *Nfat1* ^−/−^ mice were purchased from Cyagen Biosciences Inc. Cx3cr1^CreERT2^ mice (021160, B6.129P2(Cg)‐*Cx3cr1^tm2.1(cre/ERT2)Litt^* ^/WganJ^), *Tet2* ^−/−^ knockout mice (B6(Cg)‐*Tet*2^tm1.2Rao/J^), and *Cx3cr1^Gfp^* mice (005582, B6.129P2(Cg)‐*Cx3cr1^tm1Litt^*/J ) were obtained from Jackson Lab (Bar Harbor, ME, USA). H11‐LSL‐Myc (C57BL/6*‐Igs2^em1(CAG‐LSL‐Myc)Smoc^*) mice were purchased from Shanghai Model Organisms Center, Inc. (Shanghai, China). *Cx3cr1^Cre^* and *Aldh1l1^Gfp^* mice were kindly provided by Dr. Jiawei Zhou (Institute of neuroscience, Chinese academy of sciences) and Dr Tianming Gao (Department of Neurobiology, School of Basic Medical Sciences, Southern Medical University, Guangzhou, China). H11‐LSL‐Myc::Cx3cr1^CreERT2^ mice were produced by crossing H11‐LSL‐Myc hybrid mice with Cx3cr1^CreERT2^ mice. Animals were housed in the Experimental Animal Center of Nantong University with a 12 h light/dark cycle and had food and water available ad libitum. The animal study was reviewed and approved by the Ethics Committee of Nantong University (S20190128‐406) and conducted according to the Nantong University IACUC approved protocol and performed following the guidelines of the International Association for the Study of Pain.

To produce an SNL, animals were anesthetized with isoflurane and the L4 transverse process was removed to expose the L3 and L4 spinal nerves. The L4 spinal nerve was then isolated and tightly ligated with 6‐0 silk thread. For sham operations, the L4 spinal nerve was exposed but not ligated. To produce chronic inflammatory pain, CFA (10 *µ*L, Sigmal‐Aldrich, St Louis, MO, USA) was injected intraplantar under anesthetization with isoflurane.

### Nfat1^−/−^ and Nfat1^fl/fl^ Mice Generation by CRISP/Cas9 Technology

The *Nfat1* gene (NCBI Reference Sequence: NM_010899.3; Ensembl: ENSMUSG00000027544) is located on mouse chromosome 2. Exon 2–3 was selected as the target site, covering 43.4% of the coding region. Cas9 and gRNA were coinjected into fertilized eggs to produce a knockout (KO) mouse. The pups were genotyped by PCR followed by sequencing analysis (primer: 5'‐CCT GAC GTG AAA TAT CCT CAG TTG‐3'). The effective KO region's size covers 5407 bp that does not contain any other known genes (Figure S1, Supporting Information).

*Nfat1^fl/fl^* mice were generated by the CRISPR/Cas9 system‐mediated homologous recombination (Biocytogen, Beijing, China). Zygote pronuclear microinjection was performed to produce transgenic mice with a mixture of Cas9/sgRNA and a donor vector containing exon 5 flanked by 2 loxP sites and 2 homology arms of ≈1500 bp. Next, 402 injected zygotes were transferred to pseudopregnant female mice, and 6 founders were identified by Southern blot from 27 pubs. Founders were then bred with C57Bl6/J mice to produce F1 heterozygous mice (Figure S3, Supporting Information). The deletion of exon 5 results in a 573 aa (514 native aa plus 59 frame‐shift aa) truncated protein. *Cx3cr1* ^cre^::Nfat1^fl/fl^ mice were produced by crossing *Nfat1^fl/fl^* with Cx3cr1^Cre^ mice.

### DNA Extraction and Genotyping

A small piece of the ear was cut, and DNA was extracted with the phenol‐chloroform method. PCR was performed to identify WT or mutant mice. For PCR amplification, ≈500 ng DNA was used in a 30 µL reaction volume containing 15 µL 2× Taq PCR MasterMix (Vazyme) and 1 µm primers. The sequences of the primers are listed in Table S1 of the Supporting Information. The extracted DNA and primers were denatured initially at 94 °C for 4 min followed by 25 cycles at 94 °C for 30 s, 60 °C for 30 s, 72 °C for 50 s, and a final extension at 72 °C for 5 min. Amplicons were separated using 1.5% agarose gel, stained with GelRed, and photographed with GelDoc‐It Imaging System (UVP).

### Drugs and Administration

siRNAs targeting *Nfat1* (5′‐GCU AUG AGA AGA UCG UAG GTT‐3′) or *c‐Myc* (5′‐CCU UCA UCA AGA ACA UCA UTT‐3′) and scrambled negative control siRNA (NC siRNA, 5′‐UUC UCC GAA CGU GUC ACG UTT‐3′) were obtained from GenePharma Company (GenePharma, Shanghai, China). The peptide 11R‐VIVIT (RRR RRR RRR RRG GGM AGP HPV IVI TGP HEE) was purchased from Bio‐Techne (Tocris Bioscience, Sussex, UK). 5‐Bromo‐2′‐deoxyuridine (BrdU, B5002‐1G), tamoxifen (TAM, T5648), and castor oil (Cremophor EL) were purchased from Sigma‐Aldrich (St. Louis, MO, USA). For siRNA treatment, each mouse was injected with 10 µL siRNA solution with the ratio of 5 µg siRNA to 0.6 µL transfection reagent (R0541, Thermo Scientific, Waltham, MA, USA) in 5% glucose. siRNA or 11R‐VIVIT was intrathecally (i.t.) injected by lumbar puncture under anesthetization with isoflurane (3% isoflurane). BrdU was i.p. injected at 50 mg kg^−1^ body weight twice daily for 3 consecutive days. TAM or castor oil was i.p. administered once a day for 4 consecutive days.

### Real‐Time qPCR for mRNAs

For mRNA detection, the total RNA of the spinal cord or cultured cells was extracted using Trizol reagent (Invitrogen). 1 *µ*g of total RNA was reverse transcribed using an oligo(dT) primer according to the manufacturer's protocol (Vazyme). Quantitative PCR analysis was performed in the Real‐time Detection System by AceQ qPCR SYBR Green Master Mix (Vazyme). Primer sequences were listed in Table S2 of the Supporting Information. The PCR amplifications were performed at 95 °C for 3 min, followed by 40 cycles of thermal cycling at 95 °C for 10 s and 60 °C for 30 s. GAPDH was used as an endogenous control to normalize differences for mRNA detection. Melt curves were performed on completion of the cycles to ensure that nonspecific products were absent. Quantification was performed by normalizing Ct (cycle threshold) values with GAPDH Ct (mRNA) and analyzed with the 2‐^ΔΔCT^ method.

### Western Blot

Animals were transcardially perfused with 0.9% NaCl. The L4 spinal cord dorsal horn was dissected and were homogenized in a RIPA lysis buffer containing protease and phosphatase inhibitors (Roche). Protein concentrations were determined by BCA Protein Assay (Thermo Fisher Scientific). Protein samples (30 µg) were separated on SDS‐PAGE gel and transferred to PVDF membrane. The membranes were blocked with 5% milk and incubated overnight at 4°C with antibodies against NFAT1 (mouse, 1:1000, Abcam, catalog #Ab2722; Rabbit, 1:200, Cell Signaling Technology, catalog #4389S) and c‐MYC (mouse, 1:250, Santa Cruz, catalog #sc‐40). For loading control, the membranes were incubated with GAPDH antibody (mouse, 1:20 000, Sigma‐Aldrich, catalog #MAB374). Then these membranes were incubated with IRDye 800CW Goat Anti‐Mouse IgG (H + L) for 2 h at room temperature and displayed through Odyssey CLx Imaging System (LI‐COR). Specific bands were evaluated by predicted molecular size, and the intensity of selected bands was analyzed by ImageJ software.

### Immunohistochemistry

Animals were deeply anesthetized with isoflurane and perfused through the ascending aorta with 0.01 m PBS followed by 4% paraformaldehyde in 0.01 m PB. After the perfusion, the L4 spinal cord segment was removed and postfixed in the same fixative overnight. Spinal cord sections (30 µm, freefloating) were cut in a cryostat and processed for immunofluorescence. The sections were first blocked with 5% donkey serum for 2 h at room temperature, then incubated overnight at 4 °C with the following primary antibodies: NFAT1 (Rabbit, 1:50, Cell Signaling Technology, catalog #4389S), glial fibrillary acidic protein (GFAP, mouse, 1:10 000, Sigma‐Aldrich, catalog #MAB360), NeuN (mouse, 1:1000, Sigma‐Aldrich, catalog #MAB377), IBA‐1 (goat, 1:1000, Abcam, catalog #ab5607, for double staining with NFAT1), IBA‐1 (rabbit, 1:3000, Wako, catalog #019‐19741), c‐Fos (goat, 1:1000, Santa Cruz, catalog #sc‐52‐G), pp38 (rabbit, 1:1000, Cell Signaling Technology, catalog #9211S), Ki67 (rabbit, 1:250, Abcam, catalog #ab16667), BrdU (rat, 1:200, Abcam, catalog #ab6326), and c‐MYC (mouse, 1:250, Santa Cruz, catalog #sc‐40). The sections were then incubated for 2 h at room temperature with Cy3‐ or Alexa fluor 488‐ or Dylight 550‐conjugated secondary antibodies (1:1000; Jackson ImmunoResearch). For double immunofluorescence, sections were incubated with a mixture of primary antibodies from different species followed by a mixture of secondary antibodies. The stained sections were examined with a Leica SP8 Gated STED confocal microscope (Leica Microsystems, Wetzlar, Germany). The signal intensity of IBA‐1 and the counting of Ki67^+^ and BrdU^+^ cells were analyzed using ImageJ software. Three to four slices/mouse were counted with 3 mice per group.

### In Situ Hybridization

In situ probe synthesis for *Tet2* was made using Digoxigenin RNA Labeling Kit (SP6/T7, Roche Diagnostics, Mannheim, Germany). The template fragments of *Tet2* were amplified by PCR using primers (forward: 5′‐CGG GAT CCC GCC ATC ATG TTG TGG GAC GGA‐3′ and reverse: 5′‐ACG AGC TCG CGG TTG TGC TGT CAT TTG T‐3′) and subcloned into pSPT18 at the BamH I and Sac I sites. Digoxigenin (DIG)‐labeled RNA antisense and sense probes for the *Tet2* by in vitro transcription with SP6 (sense)/T7 (antisense) RNA polymerase. Digoxigenin‐labeled *Tet2* mRNA probe was detected with streptavidin–biotin complex (SABC‐FITC) kit (BOSTER, Wuhan, China). In brief, the spinal cord sections (14 µm) were treated with 30% H_2_O_2_ and methanol (1:50) for 30 min at room temperature. After being washed with DEPC‐treated ultrapure water, the sections were prehybridized at 42 °C for 4 h at room temperature and hybridized with the DIG‐labeled probe (1 µg mL^−1^) in hybridization buffer at 42 °C overnight. After being washed by sodium chloride–sodium citrate buffer, sections were then incubated in blocking solution at 37 °C for 30 min and in mouse anti‐DIG‐biotin for 60 min at room temperature, washed with PBS (0.01 m, pH 7.4), and then incubated in the streptavidin–biotin complex‐FITC reagent for 30 min at 37 °C. To further identify the cell types expressing *Tet2* in the spinal cord, the sections under in situ hybridization were further incubated overnight using primary rabbit antibodies against NeuN and IBA‐1 and then further incubated with Cy3‐conjugated secondary antibodies for 2 h at room temperature. The signals were detected with Leica SP8 confocal microscope.

### Single‐Cell RT‐PCR Assay

Single‐cell RT‐PCR was performed as described previously.^[^ ^11^, ^48^ ^]^ GFP‐labeled microglial cell and astrocytes were extracted from *Cx3cr1^Gfp^* mice and *Aldh1l1^Gfp^* mice, respectively. The contents of neurons are harvested from the lamina II of spinal cord slices from C57Bl/6 mice. Reverse transcriptions were performed using an Invitrogen SuperScript Reagent Kit (Invitrogen, CA, USA). The first step of the reverse transcription system was performed at 37 °C for 40 min and then at 80 °C for 10 min to remove the genomic DNA. The second reaction was then performed at 50 °C for 50 min and 70 °C for 15 min. Single‐cell PCR was performed as a nested PCR. The external primers were used for the primary amplification, and internal primers were used for the following secondary amplification. The sequences of nested PCR primers are depicted in Table S3 of the Supporting Information. All PCR products were visualized using agarose gel (2.5%) electrophoresis with Gel‐Red.

### Methylated DNA Immunoprecipitation and High‐Throughput Sequencing (MeDIP‐seq)

MethPrimer (http://www.urogene.org/methprimer/) and CpG Islands Track from the UCSC browser were used to predict the CpG islands of *Nfat1*.^[^ ^49^ ^]^ DNAs obtained from spinal dorsal horn tissues were fragmented to ≈200–900 bp fragments using a BioRuptor machine (Diagenode, Belgium). According to the instruction, illumina‐supplied universal adapters were ligated to the fragmented genomic DNA using Genomic DNA Sample Kit (Illumina San Diego, catalog #FC‐102‐1002). The ligated DNA fragments were further immunoprecipitated by the anti‐5‐methylcytosine antibody (Diagenode, Denville, USA). PCR amplification was performed to enrich precipitated fragments, and then gel purification was used to extract ≈300–1000 bp DNA fragments. Sequencing was performed on Illumina HiSeq 2000 using TruSeq Rapid SBS Kit (Illumina, catalog #FC‐402‐4001,). After sequencing images generated, the stages of image analysis and base calling were performed using Off‐Line Basecaller software (OLB V1.8, Illumina). After passing the Solexa CHASTITY quality filter, the clean reads were aligned to the mouse genome (UCSC MM10) using BOWTIE software (V2.1.0).

### Methylation‐Specific PCR

According to the previous work,^[^ ^23^, ^28^ ^]^ the genomic DNA was extracted from the spinal cord through a QIAamp DNA Mini Kit (Qiagen, CA, USA) . Sodium bisulfite conversion of genomic DNA and recovery were performed with the EpiTect Bisulfite Kit (Qiagen). MSP was performed using EpiTect Master Mix (Qiagen) with methylation‐specific or unmethylation‐specific primers (Table S4, Supporting Information). The PCR products were analyzed by electrophoresis. The methylation levels of the *Nfat1* promoter were evaluated through densitometric analysis of MSP products (ratio of methylated products to unmethylated products).

### Bisulfite Sequencing

The genomic DNA extraction and bisulfite conversion were performed in the same manner as described in the MSP method. Then, PCR was performed to amplify the CpG island fragment of the *Nfat1* promoter using the primers shown in Table S4 of the Supporting Information. The PCR products were purified with the QIAquick Gel Extraction Kit (Qiagen). The eluted DNA fragments were cloned into pGEM‐T Easy Vector (Promega) for sequencing. Ten colonies for each mouse were randomly chosen for sequencing.

### MeDIP‐ and Hydroxymethylated DNA Immunoprecipitation (hmeDIP)‐qPCR

Experiments were performed as described.^[^ ^50^ ^]^ Genomic DNA was extracted from the lumbar 4–5 spinal dorsal horn in ipsilateral and sonicated to a size of 200–800 bp. DNA fragments were denatured and immunoprecipitated with mouse monoclonal antibody against 5mC (Abcam, Catalog #ab10805), 5hmC (Abcam, catalog #ab214728), or normal mouse IgG (Abcam, catalog #ab18443) at 4 °C overnight. Precipitated DNA was cleared with magnetic Protein G beads (Invitrogen). qPCR was used to amplify the enriched DNA and the PCR products were separated by agarose gel electrophoresis. Primers used for MeDIP‐ and hMeDIP‐qPCR are listed in Table S4 of the Supporting Information.

### Nfat1 Promoter Activity Analysis

The *Nfat1* promoter‐reporter cloned into the pCpG‐free basic reporter vector (InvivoGen) was either methylated by incubation with S‐adenosyl methionine or unmethylated in the presence or absence of CpG methylase M.SssI (ThermoFisher Scientific). The methylated or unmethylated pCpG‐free‐*Nfat1*‐Lucia luciferase reporter plasmid was transfected into HEK293 cells by Lipofectamine 3000 (Invitrogen). The secreted coelenterazine luciferase activity in the medium was detected 48 h later using the Dual‐Luciferase Assay System (Promega) following the instructions. 20 *µ*L of the medium samples was subjected to luciferase assay (BioTek, VT, USA).

### Spinal Slice Preparation

The lumbar spinal cord was carefully removed from WT or *Nfat1* ^−/−^ mice (4–6 weeks) under urethane anesthesia (1.5–2 g kg^−1^, i.p.) and placed in preoxygenated (saturated with 95% O_2_ and 5% CO_2_) ice‐cold sucrose artificial CSF (aCSF) solution. The sucrose aCSF contained the following (in mm): 234 sucrose, 3.6 KCl, 1.2 MgCl_2_, 2.5 CaCl_2_, 1.2 NaH_2_PO_4_, 12 glucose, and 25 NaHCO_3_. The pia‐arachnoid membrane was gently removed from the section. The portion of the lumbar spinal cord was identified by the lumbar enlargement and large dorsal roots. The spinal segment was placed in a shallow groove formed in an agar block and then glued to the button stage of a VT1000S vibratome (Leica). Transverse slices (450 µm) were cut in the ice‐cold sucrose aCSF, incubated in Krebs’ solution oxygenated with 95% O_2_ and 5% CO_2_ at 34 °C for 30 min, and then allowed to recover 1–2 h at room temperature before the experiment. The Krebs’ solution contained the following (in mm): 117 NaCl, 3.6 KCl, 1.2 MgCl_2_, 2.5 CaCl_2_, 1.2 NaH2PO_4_, 25 NaHCO_3_, and 11 glucose.

### Patch‐Clamp Recordings in Spinal Slices

The voltage‐clamp recordings were made from neurons in outer lamina II of the dorsal horn. The slice was continuously superfused (3–5 mL min^−1^) with Krebs’ solution at room temperature and saturated with 95% O_2_ and 5% CO_2_. Individual neurons were visualized under a stage‐fixed upright infrared differential interference contrast microscope (BX51WI, Olympus) equipped with a 40× water‐immersion objective. The patch pipettes were pulled using a Flaming micropipette puller (P‐97, Sutter Instruments), and had initial resistance of 5–10 MΩ when filled with the internal pipette solution contained the following (in mm):120 CsMeSO_3_, 2 NaCl, 20 HEPES, 5 tetraethylammonium‐Cl, 2.5 Na_2_ATP, 0.4 EGTA, 0.3 GTP‐Tris, and 2.5 mm QX‐314 (pH 7.2‐7.4, adjusted with CsOH). Membrane voltage and current were amplified with a multiclamp 700B amplifier (Molecular Devices). Data were filtered at 2 kHz and digitized at 10 kHz using a data acquisition interface (1440A, Molecular Devices). A seal resistance (≈2 GΩ) and an access resistance (35 MΩ) were considered acceptable. The cell capacity transients were canceled by the capacitive cancelation circuitry on the amplifier. After establishing the whole‐cell configuration, the membrane potential was held at 70 mV for recording sEPSCs. The sIPSCs were recorded with the membrane voltage held at 0 mV. Data were stored with a personal computer using pClamp10.0 software and analyzed with Mini Analysis (Synaptosoft 6.0). Those cells that showed ≈10% changes from the baseline levels were regarded as responsive to the presence of drugs.

### RNA Isolation and Microarray

Total RNA was isolated from the L4 spinal cord dorsal horn at 5 days after SNL or sham operation using the TRIzol reagent (Invitrogen, Carlsbad, CA). Three treatment groups were set up in this experiment: WT‐Sham, WT‐SNL, and *Nfat1* ^−/−^‐SNL. Each group had two repeats. Each RNA sample was a mixture of mRNAs from three mice under the same treatment. Following isolation, RNA was further purified with a NucleoSpin RNA clean‐up kit (Macherey‐Nagel, Germany). The concentration and yield of RNA samples were determined by a NanoDrop ND‐2000 Spectrophotometer (Thermo Scientific, Waltham, MA). Gene expression profiles of the L4 spinal cord dorsal horn were assessed with Agilent SurePrint G3 Mouse GE 8 × 60K Microarray Kit (G4852A) by CapitalBio Corporation (Beijing, China). Expression data were submitted to the GEO database (GSE184052).

### Plasmids Preparation

Promoter sequences (from −1000 to + 200 bp relative to TSS) of *Itgam*, *Tnf*, *Il‐1β*, and *c‐Myc* promoters were obtained by using data from the RefSeq database (NM_008401, NM_013693, NM_008361, and NM_001177354). JASPAR websites predicted NFAT1 binding sites on the above promoters. Each NFAT1 binding site on the above promoters was mutated and synthesized separately by Shenggong Bioengineering Technology Limited (Shanghai, China). Next, the WT and mutant promoters were cloned into the pGL3‐basic vector (Promega, Madison, WI) to drive luciferase reporters. NFAT1 overexpression plasmid was obtained from Addgene (HA‐NFAT1(4‐460)‐GFP, catalog #11107). Plasmid pCDNA3.1 was obtained from Invitrogen (Carlsbad, CA).

### Dual‐Luciferase Reporter Assays

HEK293 cells were plated in 12‐well plates and transfected using Lipofectamine 3000 (Thermo Fisher Scientific, Carlsbad, CA) with the indicated different combinations of luciferase reporter vectors (250 ng), the Renilla reporter vector (pRL‐TK, 20 ng), NFAT1 overexpressing vector (250 ng), or empty control vector (250 ng). Luciferase assays were performed 48 h after transfection. Activities of firefly and Renilla luciferase were measured using the Dual‐Glo Luciferases Assay System (Promega, Madison, WI) by a Synergy 2 multidetection microplate reader (BioTek, VT, USA) as described previously.^[^ ^23^, ^28^ ^]^

### ChIP Quantitative PCR (ChIP‐qPCR)

ChIP was performed using the Simple ChIP Enzymatic Chromatin IP Kit (#9003, Cell Signaling Technology) according to the manufacturer's protocol.^[^ ^23^, ^28^ ^]^ In brief, single‐cell suspensions of spinal dorsal horn tissues were prepared using a glass tissue homogenizer. Then, cells were crosslinked with formaldehyde (final concentration 1%) for 10 min, and the cross‐linking was quenched by the addition of glycine (final concentration 0.125 m). Cells were then lysed, chromatin was harvested and fragmented using micrococcal nuclease digestion, and nuclear membranes were broken by sonication. The disposed chromatin was subjected to immunoprecipitation with normal mouse IgG antibody (Cell Signaling Technology, catalog #5415) as the negative control, rabbit histone H3 antibody (Cell Signaling Technology, catalog #14269) as the positive control, and NFAT1 mouse monoclonal antibody (Abcam, catalog #ab2722). The ChIP‐enriched DNA samples were quantified by quantitative ChIP‐PCR using specific primer pairs (Table S5, Supporting Information), and the PCR products were visualized by agarose gel electrophoresis.

### Behavioral Analysis

Animals were habituated to the testing environment daily for at least 2 days before baseline testing. All the behavioral experimenters were done by individuals that were blinded to the treatment or genotypes of the mice. i) Tail immersion test. The temperature of the water was set at 48, 50, or 52 °C. Gently stroking the tail of the mouse so that its tail droops naturally into the water at different temperatures and immerse it into a length of ≈1 cm. The tail‐flick latency was recorded. The cutoff was set at 20 s to avoid potential injury. ii) Hargreaves test. The animals were placed in a plastic box on a glass plate and allowed 30 min for habituation. Heat sensitivity was tested by radiant heat using the Hargreaves apparatus (IITC model 390 Analgesia Meter, Life Science), which was expressed as paw withdrawal latency. The latency baseline was adjusted to 8–14 s with a maximum of 20 s as cut off to prevent potential injury. The latencies were averaged over three trials, separated by a 5 min interval. iii) Von Frey test. The animals were put in boxes on an elevated metal mesh floor and allowed 30 min for habituation before the examination. The plantar surface of the hind paw was stimulated with a series of von Frey hairs with logarithmically incrementing stiffness (0.02–2.56 g, Stoelting, Wood Dale, IL), presented perpendicular to the plantar surface (2–3 s for each hair). The 50% paw withdrawal threshold was determined using Dixon's up–down method. On days 1, 3, 7, and 14 after surgery, animals are evaluated to corroborate the presence of mechanical allodynia. iv) Rota‐rod test. Mice were trained on the rota‐rod for 5 min at a speed of 10 rpm till the mice no longer fell off it. For testing, the speed was set at 10 rpm for adaptation with 5 min and subsequently accelerated to 80 rpm in 10 min. The time taken for mice to fall after the beginning of the acceleration was recorded. v) Spontaneous pain test. On the day of behavioral testing, mice were individually placed in small plastic chambers and allowed at least 30 min for habituation. Formalin (5%, 10 *µ*L) was injected into a hind paw. Immediately after the injection, mice were returned to the chambers and recorded for 45 min. The spontaneous pain behaviors were measured by counting the time (in seconds) mice spent licking, lifting, and flinching the affected paw every 5 min.

### Quantification and Statistics

All sample sizes and experimental designs were based on previously published data from the lab and similar experiments in the field. All data were expressed as mean ± SEM. For the analysis of IBA‐1 immunoreactivity, the dorsal horn images were captured, and a numerical value of the intensity was calculated with a computer‐assisted imaging analysis system (ImageJ). The intensity of the background was subtracted in each section. For Western blotting, the density of specific bands was measured with ImageJ. All statistical tests were conducted using two‐tailed hypothesis testing. Normality of data was assessed using Kolmogorov–Smirnov test. Rank transformation test was used when the data were not normally distributed (mechanical allodynia behavior). For the immunostaining data, Western blotting, and electrophysiological data, if only two groups were applied, Student's *t*‐test was used; if more than three groups were applied, one‐way ANOVA was used. If the differences were significant, post hoc Bonferroni's test was applied to compare the difference between every two groups. For the behavioral test, two‐way repeated‐measures (RM) ANOVA was used. If the differences were significant, post hoc Bonferroni's test was applied to compare values at different time points. All statistical analyses were performed using GraphPad Prism 6 (GraphPad Software, Inc.). *P* < 0.05 (two‐tailed) was considered statistically significant. * or # *P* < 0.05, ** or ## *P* < 0.01, *** or ### *P* < 0.001. The *N* for each experiment is described in the figure legends of Figures 1, 2, 3, 4, 5, 6, 7, 8.

## Conflict of Interest

The authors declare no conflict of interest.

## Author Contributions

B.‐C.J. and T.‐Y.D. contributed equally to this work. B.‐C.J. performed the gene array analysis, DNA methylation, and analyzed the data. T.‐Y.D., B.‐C.J., and C.‐Y.G. performed quantitative PCR, behavioral tests, Western blotting, and immunostaining. D.‐L.C. performed the single‐cell PCR. X.‐B.W. and W.‐L.S. conducted the electrophysiological recording. X.‐H.B. and M.J. participated in the immunostaining and behavioral experiments. L.‐J.W. participated in the manuscript's preparation. Y.‐J.G. and B.‐C.J. designed the experiments. Y.‐J.G. and B.‐C.J. coordinated and supervised the project and wrote the manuscript.

## Supporting information

Supporting Information

## Data Availability

The data that support the findings of this study are available from the corresponding authors upon reasonable request.
