# Supplementary material for: NFAT1 Orchestrates Spinal Microglial Transcription and Promotes Microglial Proliferation via c‐MYC Contributing to Nerve Injury‐Induced Neuropathic Pain
Source: Adv Sci (Weinh). 2022 Jul 27;9(27):2201300. doi: 10.1002/advs.202201300 (PMC9507349; doi:10.1002/advs.202201300)
Supplement: Supplementary file 1 — Supporting Information [file ADVS-9-2201300-s001.pdf]

ADVANCED SCIENCE
Open Access

Supporting Information

for *Adv. Sci.*, DOI 10.1002/advs.202201300

NFAT1 Orchestrates Spinal Microglial Transcription and Promotes Microglial Proliferation via c-MYC Contributing to Nerve Injury-Induced Neuropathic Pain

Bao-Chun Jiang, Ting-Yu Ding, Chang-Yun Guo, Xue-Hui Bai, De-Li Cao, Xiao-Bo Wu, Wei-Lin Sha, Ming Jiang, Long-Jun Wu and Yong-Jing Gao**

NFAT1 orchestrates spinal microglial transcription and promotes microglial proliferation via c-MYC contributing to nerve injury-induced neuropathic pain

Bao-Chun Jiang,[*] Ting-Yu Ding, Chang-Yun Guo, Xue-Hui Bai, De-Li Cao, Xiao-Bo Wu, Wei-Lin Sha, Ming Jiang, Long-Jun Wu, Yong-Jing Gao[*]

Inventory of supplementary materials submitted:

I. Supplementary Figures (7)

II. Supplementary Tables (5)

Supplementary figures

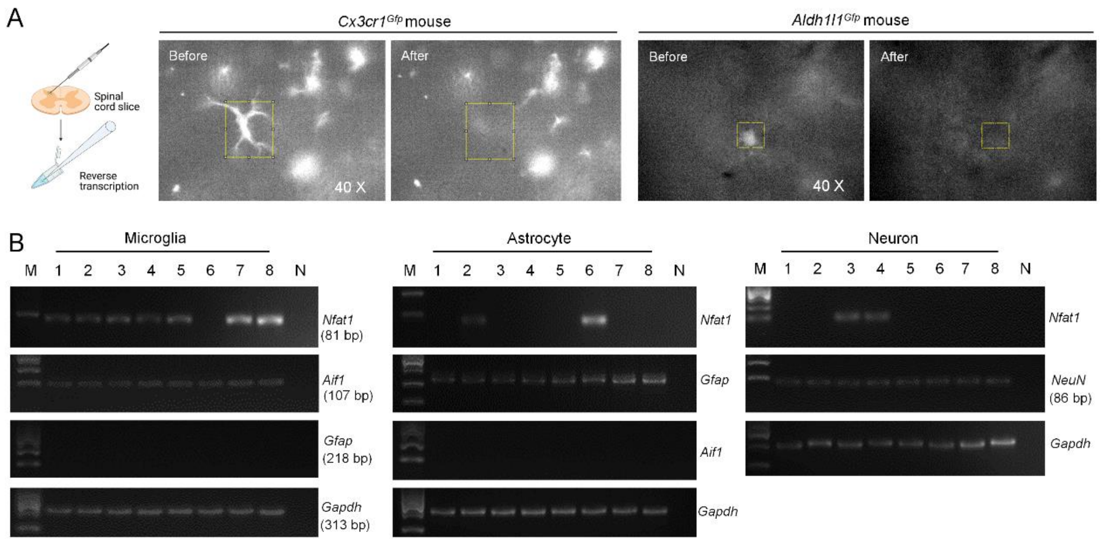

Figure S1. Single-cell nested PCR for *Nfat1* in the spinal dorsal horn from SNL treated mice. (A) Schematic of the process of single-cell PCR (Left panel). The content of a microglia (middle panel) or an astrocyte (right panel) was sucked into glass pipettes. (B) Single-cell PCR analysis showing the expression of *Nfat1* in 7/8 microglia, 1/8 astrocytes, and 2/8 neurons in the dorsal horn of spinal cord slices.

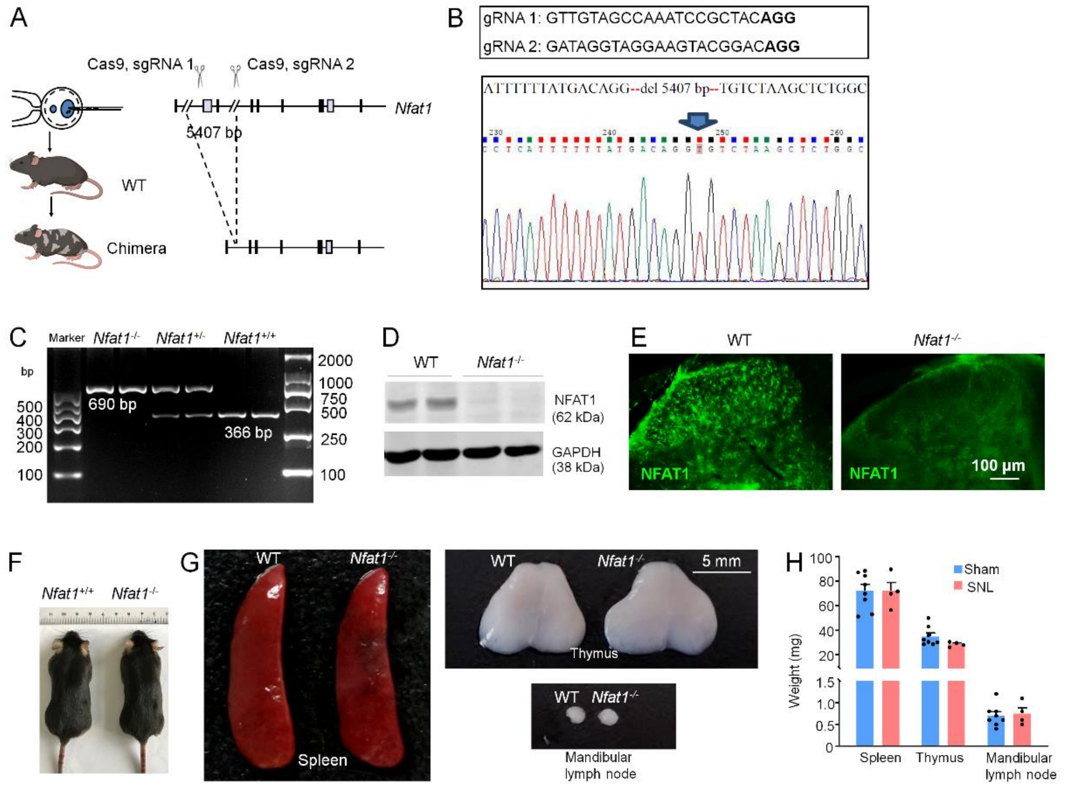

Figure S2. Identification of *Nfat1*$^{-/-}$ mice. (A) Schematic diagram shows the strategy for generating *Nfat1*$^{-/-}$ mice by the CRISPR-Cas9 system. (B) Sanger sequencing shows the deletion of 5407 bp fragment of *Nfat1* gene. (C) Genotyping of *Nfat1*$^{-/-}$, *Nfat1*$^{+/-}$ and *Nfat1*$^{+/+}$ (WT) mice. (D) Western blot shows the missing band of NFAT1 in *Nfat1*$^{-/-}$ mice. (E) Immunostaining shows the missing signal of NFAT1 in *Nfat1*$^{-/-}$ mice. (F) Photographs of WT and *Nfat1*$^{-/-}$ mice show no gross anatomy changes. (G) Gross morphologies of the dissected spleen, thymocytes, and lymph node in 8 week-old WT and *Nfat1*$^{-/-}$ mice. (H) The weight of the above immune organs is comparable between WT and *Nfat1*$^{-/-}$ mice.

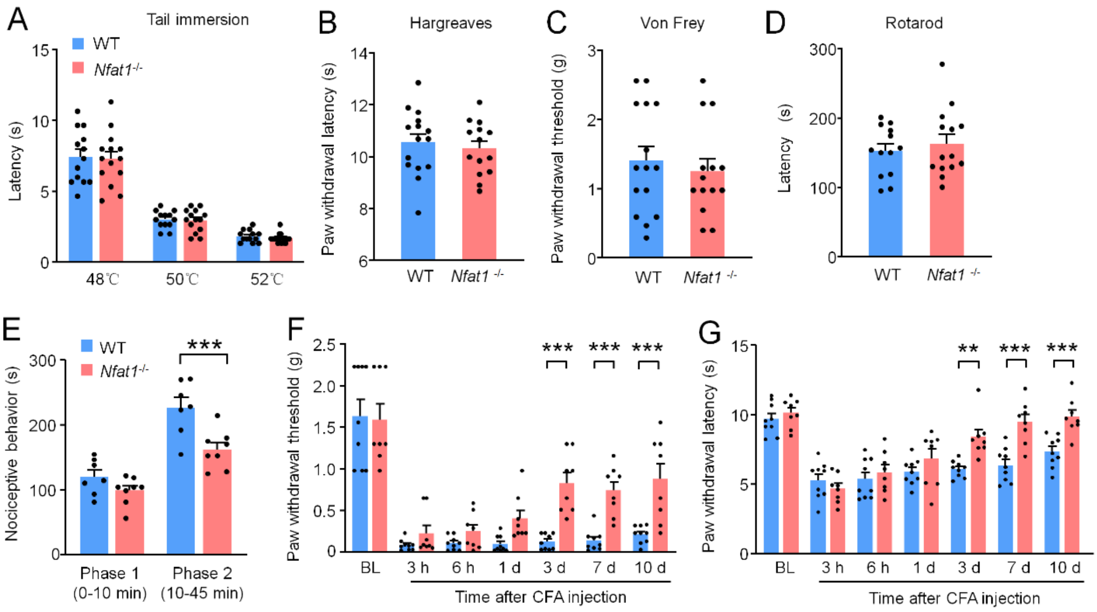

Figure S3. ***Nfat1***$^{-/-}$ **mice show normal basal pain and motor function and decreased inflammatory pain.** (A-C) Deletion of *Nfat1* does not affect the basal pain threshold. Pain threshold were detected by Tail immersion (A), Hargreaves test (B), and Von Frey test (C). (D) Deletion of *Nfat1* does not affect motor function, which was assessed by the Rota-rod test. n = 13 - 15. (E) The spontaneous pain in phase 2 induced by 5% formalin is significantly decreased in *Nfat1*$^{-/-}$ mice compared with WT mice. $F_{(1, 26)}$ = 14.44, P = 0.008, two-way RM ANOVA. n = 7-8 mice/group. (F, G) CFA-induced mechanical allodynia (F; $F_{(1, 105)}$ = 40.02, P < 0.0001, two-way RM ANOVA) and heat hyperalgesia (G; $F_{(1, 105)}$ = 30.87, P < 0.0001, two-way RM ANOVA) were reduced in *Nfat1*$^{-/-}$ mice. n = 8-9 mice/group.

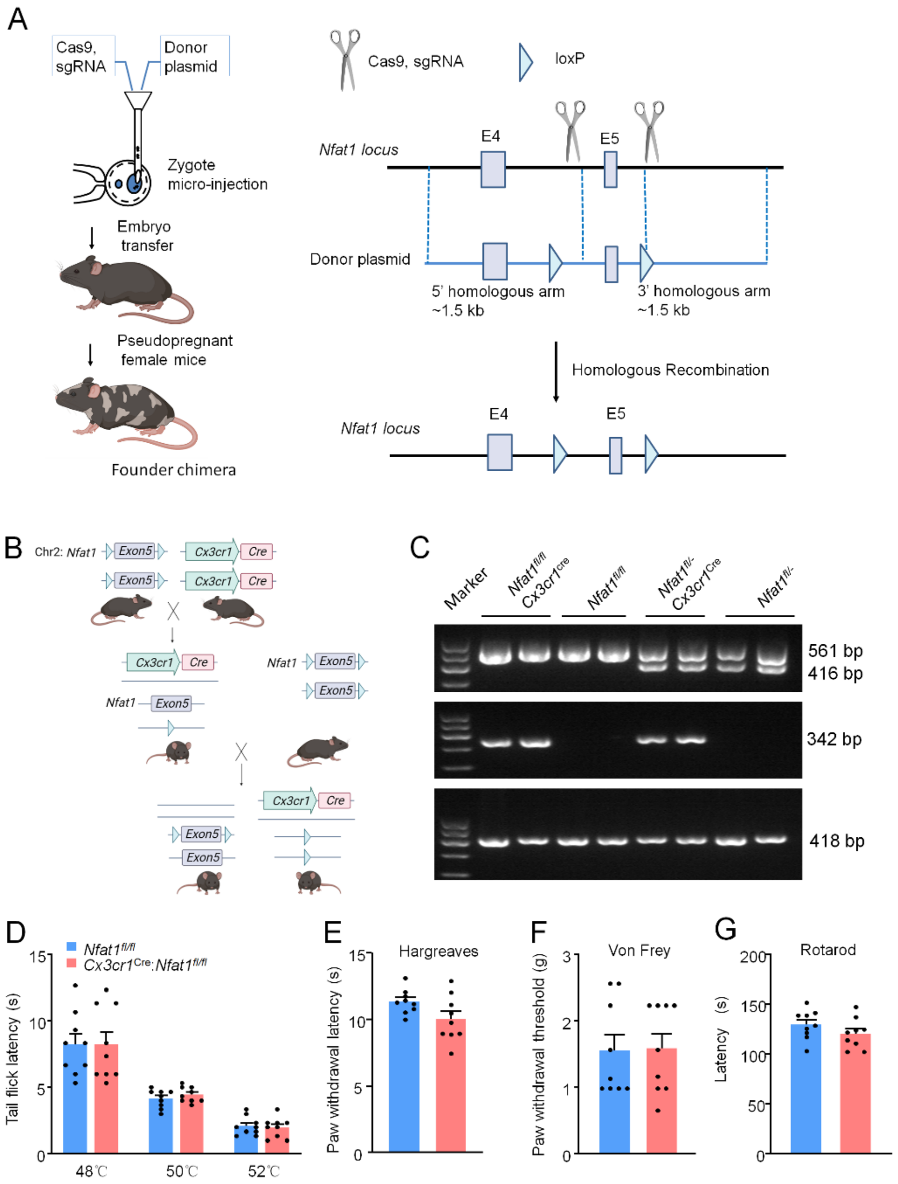

Figure S4. Generation and behavioral test of *Cx3cr1*^{Cre}:*Nfat1*$^{fl/fl}$ mice. (A) Schematic overview of the strategy to generate mice carrying *Nfat1* flox allele using the CRISPR/Cas9 system. LoxP sites are indicated as triangles, and the scissors indicate the target sites for each CRISPR/Cas9 nuclease. The donor plasmid contains 2 homology arms of ~1500 bp each flanking the loxP-floxed exon 5. (B) Schematic diagram shows the generation of microglia-specific *Nfat1* conditional knockout mice. Homozygous *Cx3cr1*^{Cre} and *Nfat1*$^{fl/fl}$ mice were crossed to obtain heterozygous *Cx3cr1*^{Cre}::*Nfat1*$^{fl/-}$ mice. These mice were further crossed to *Nfat1*$^{fl/fl}$ mice to generate heterozygous *Cx3cr1*^{Cre}::*Nfat1*$^{-/-}$ mice. (C) Genotyping of *Nfat1* WT or flox and *Cx3cr1*^{Cre} recombinase DNA. In the top panel, the 416-bp band shows the WT allele, and 561-bp band shows the floxed-*Nfat1* allele. The 342-bp band represents *Cx3cr1*^{Cre} allele, while the 418-bp band

represents WT. (D-F) Tail immersion (D) and Hargreaves test (E) were detected for thermal sensitivity and von Frey test (F) for mechanical sensitivity. (G) The Rota-rod test assessed motor function. For tail immersion latency, $F_{(1, 16)} = 0.01794$, $P = 0.8951$, two-way ANOVA followed by Bonferroni's test. For others, $P > 0.05$, Student's t-test, n = 9/group.

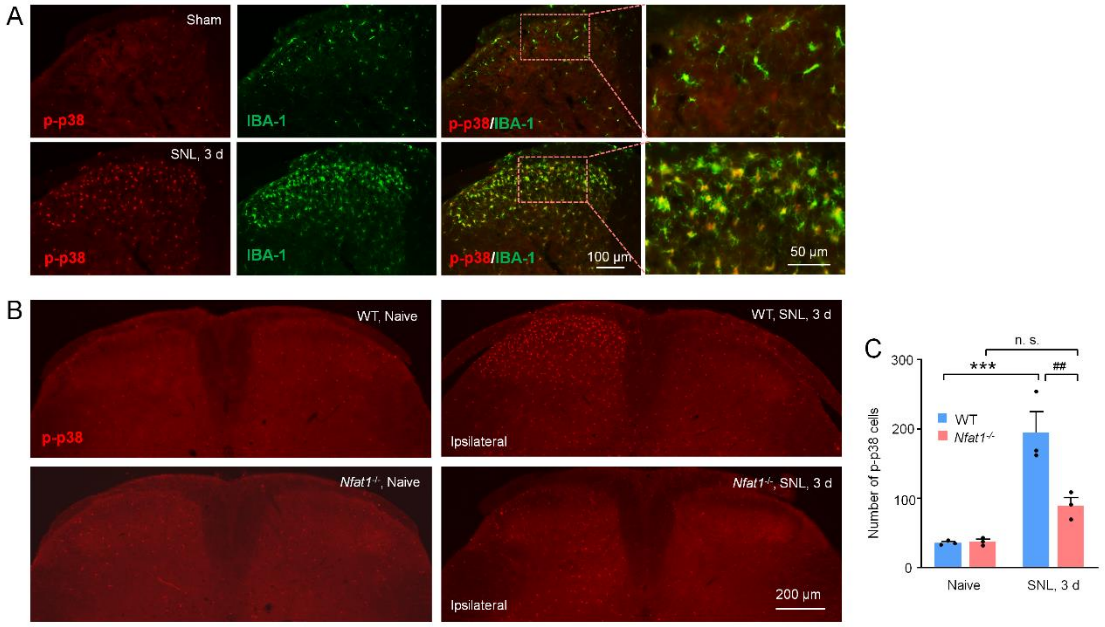

Figure S5. Deficiency of *Nfat1* reduces p38 activation. (A) Double staining of p-p38 with IBA-1 in WT mice after sham or SNL operation. (B) Immunostaining of p-p38 in the spinal cord of WT and *Nfat1*$^{-/-}$ mice. (C) The number of pp38-positive cells is dramatically increased in WT mice but not significantly increased in *Nfat1*$^{-/-}$ mice. *** P < 0.001, WT-SNL *vs.* WT-Naïve; $^{\#\#}P$ < 0.01, *Nfat1*$^{-/-}$-SNL *vs.* WT-SNL. n.s. no significance.

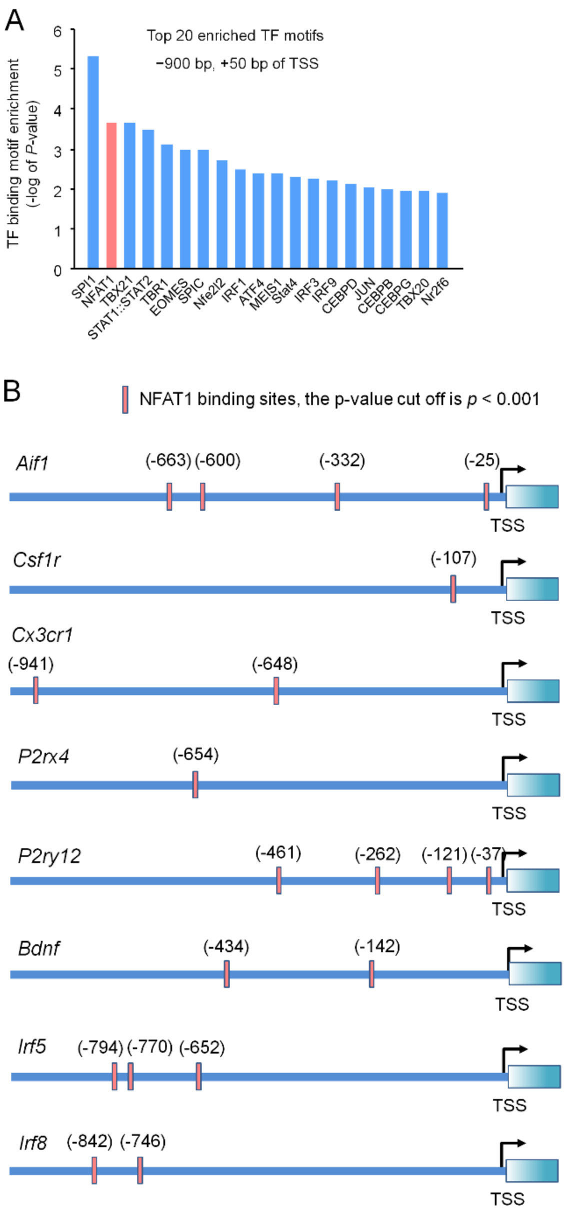

Figure S6. Identification of NFAT1 target genes. (A) TF binding motif enrichment analysis for genes significantly upregulated in the spinal cord after SNL. NFAT1 binding site is the second most significantly enriched motif. (B) The transcription binding motif of NFAT1 in *Aif1*, *Csf1r*, *Cx3cr1*, *P2rx4*, *P2ry12*, *Bdnf*, *Irf5*, and *Irf8* is predicted by the JASPAR CORE vertebrate database.

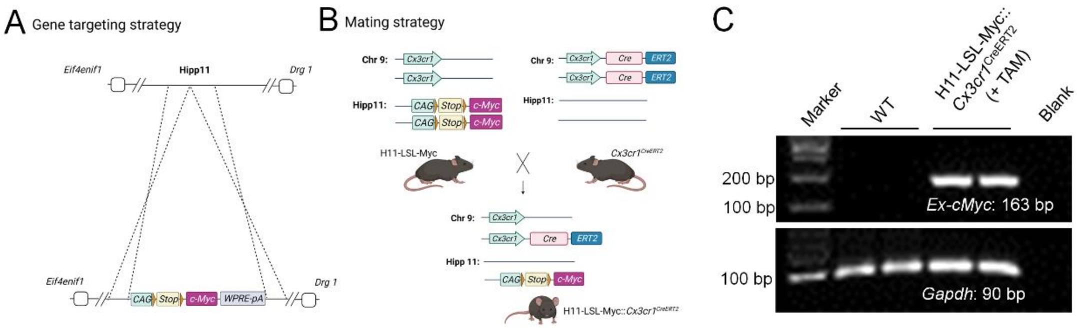

Figure S7. Overexpression of c-Myc in H11-LSL-Myc::*Cx3cr1*$^{\text{CreERT2}}$ mice. (A) Schematic illustration of the WT Hipp11 and CAG-driven loxP-STOP-loxP-c-Myc targeting construct after integration into to the Hipp11 locus on chromosome 11. (B) Schematic showing the conditional CAG-driven loxP-STOP-loxP-c-Myc (LSL-Myc) knock-in mouse model. C-Myc expression is prevented by a LoxP-stop-LoxP sequence, which can be removed by *Cre* recombinase. (C) Real-time PCR shows that the exogenous c-Myc is successfully induced by tamoxifen (TAM) in the spinal cord of H11-LSL-Myc::*Cx3cr1*^{CreERT2} double-positive mice.

Table S1. Primers for genotyping of genetic modification mice

Sequence name	Sequence	Size of PCR products (bp)
Nfat1$^{-/-}$-Com-F	5'-CCT GAC GTG AAA TAT CCT CAG TTG-3'	WT: 366<br>Hom: 690<br>Het: 690/366
Nfat1$^{-/-}$-R1	5'-ACG TAA ATG TCA AAA GGG AGG CG-3'	
Nfat1$^{-/-}$-R2	5'-ATT GTA CAG GAC AAT GGG CTT C-3'	
Nfat1$^{fl/fl}$-F	5'-ATT TCA GTG GCA ACA CAG AAG GCC A-3'	WT: 416<br>Hom: 561<br>Het: 416/561
Nfat1$^{fl/fl}$-R	5'-GCA CTT GGA CAA TCT CCT GAG TGG A-3'	
Cx3cr1^{Cre}-F1	5'-TTT TGA GTA TGA CGA TTC TGC TG-3'	WT: 0<br>Hom: 342<br>Het: 0/342
Cx3cr1^{Cre}-R1	5'-ACT AAT GGT GAC ACC GTG CTG-3'	
Cx3cr1^{Cre}-F2	5'-GAT AGT GAA ACA GGG GCA ATG-3'	WT: 0<br>Hom: 418<br>Het: 0/418
Cx3cr1^{Cre}-R2	5'-TAC CCA AGA GTT CGC CAA AC-3'	
Tet2$^{-/-}$-Com-F	5'-TCT CAG AGC AAA GAG GAC TGC-3'	WT: 536<br>Hom:200<br>Het: 536 /200
Tet2$^{-/-}$-R1	5'-AGC TGA TGG AAA ATG CAA GC-3'	
Tet2$^{-/-}$-R2	5'-GCC ACT TTA GAA GCC TAT TGG A-3'	
Cx3cr1^{CreERT2}-Com-F	5'-AAG ACT CAC GTG GAC CTG CT-3'	WT: 695<br>Hom: 300<br>Het:300/695
Cx3cr1^{CreERT2}-R1	5'-AGG ATG TTG ACT TCC GAG TTG-3'	
Cx3cr1^{CreERT2}-R2	5'-CGG TTA TTC AAC TTG CAC CA-3'	
H11-LSL-*cMyc*-F1	5'-ATA AGC CAT TCT CCA TTT CAT AA-3'	WT: 428<br>Hom: 434<br>Het: 428/434
H11-LSL-*cMyc*-R1	5'-CCC CTT GTT CCC TTT CTG C-3'	
H11-LSL-*cMyc*-F2	5'-CTC CCC CGT GCC TTC CTT GAC-3'	
H11-LSL-*cMyc*-R2	5'-TTT GCC TTT GTT ACC TGT TCC ATC-3'	

Table S2. Primer sets for qPCR

Gene	Primer Sequence	Size of PCR products (bp)
Nfat1	5'-CGC ACG CCT TCT ACC AAG TA-3'<br>5'-GTC GAT GGT GGC TCT CAT GT-3'	134
Nfat2	5'-GGA GAG TCC GAG AAT CGA GAT-3'<br>5'-TTG CAG CTA GGA AGT ACG TCT-3'	102
Nfat3	5'-GAG CTG GAA TTT AAG CTG GTG T-3'<br>5'-CAT GGA GGG GTA TCC TCT GAG-3'	101
Nfat4	5'-TGC TCA ACT TCC GTC AAG GAC-3'<br>5'-GAT GTG GTA AGC CAA GGG ATG-3'	134
Dnmt3b	5'-CTG TCC GAA CCC GAC ATA GC-3'<br>5'-CCG GAA ACT CCA CAG GGT A-3'	120
Tet2	5'-AAG CAG CCG TCA GCC AAA T-3'<br>5'-TTC CGT GTT GGG AAA GCA TCT-3'	102
Itgam	5'-TGC TGG GGA GGT CAG AGT C-3'<br>5'-CTC ATC AAA GAA GGC ACG G-3'	139
Aif1	5'-ATG AGC CAA AGC AGG GAT T-3'<br>5'-CTT CAA GTT TGG ACG GCA G-3'	145
Cx3cr1	5'-GAG TAT GAC GAT TCT GCT GAG G-3'<br>5'-CAG ACC GAA CGT GAA GAC GAG-3'	102
Csf1r	5'-TGT CAT CGA GCC TAG TGG C-3'<br>5'-CGG GAG ATT CAG GGT CCA AG-3'	134
P2rx4	5'-ACC AGG AAA CGG ACT CTG TG-3'<br>5'-TCA CGG TGA CGA TCA TGT TGG-3'	168
P2ry12	5'-CCC TGT GCG TCA GAG ACT AC-3'<br>5'-CAA GCT GTT CGT GAT GAG CC-3'	92
Bdnf	5'-CCT CCT CCC ATT TTG GTC CC-3'<br>5'-TCT GCA AAC ACT GTT AGG CCA-3'	243
Tnf	5'-GTT CTA TGG CCC AGA CCC TCA C-3'<br>5'-GGC ACC ACT AGT TGG TTG TCT TTG-3'	175

Il-1b	5'-TCC AGG ATG AGG ACA TGA GCA C-3'<br>5'-GAA CGT CAC ACA CCA GCA GGT TA-3'	105
Irf5	5'-CCT CAG CCG TAC AAG ATC TAC GA-3'<br>5'-GTA GCA TTC TCT GGA GCT CTT CCT-3'	127
Irf8	5'-CGG GGC TGA TCT GGG AAA AT-3'<br>5'-CAC AGC GTA ACC TCG TCT TC-3'	183
c-Myc	5'-ATG CCC CTC AAC GTG AAC TTC-3'<br>5'-GTC GCA GAT GAA ATA GGG CTG-3'	78
Exogenous c-Myc	5'-TGA GGA AAC GAC GAG AAC AGT-3'<br>5'-GCA GCG TAT CCA CAT AGC GT-3'	163
Gapdh	5'-AAA TGG TGA AGG TCG GTG TGA AC-3'<br>5'-CAA CAA TCT CCA CTT TGC CAC TG-3'	90

Table S3. Primer sequences for single-cell RT-PCR

Gene	Primer Sequence	Size of PCR products (bp)
Nfat1 outer	5'-CCA CTC TCC AAT CAG TCG GG-3'<br>5'-TAC TTG GTA GAA GGC GTG CG-3'	231
Nfat1 inner	5'-ATT GAG GTC CAA CCC AAG CC-3'<br>5'-TCC TGT TGG GGC TTT GAC AG-3'	81
Aif1 outer	5'-TGG AGG GGA TCA ACA AGC AA-3'<br>5'-TGG GAA CCC CAA GTT TCT CC-3'	171
Aif1 inner	5'-TGA TGA GGA TCT GCC GTC CA-3'<br>5'-CCA GCA TTC GCT TCA AGG AC-3'	107
Gfap outer	5'-CCT CAG CCG TAC AAG ATC TAC GA-3'<br>5'-CGT CCA GAG GGA ACT AAC TAA C-3'	371
Gfap inner	5'-GCC TAT GCT AAA GGT TAG GTT GTA-3'<br>5'-AGC ACT GAA GTG AAG CAA TAG A-3'	218
NeuN outer	5'-AGA CAG ACA ACC AGC AAC TC-3'<br>5'-CTG TTC CTA CCA CAG GGT TTA G-3'	357
NeuN inner	5'-ACG ATC GTA GAG GGA CGG AA-3'<br>5'-TTG GCA TAT GGG TTC CCA GG-3'	86
Gapdh outer	5'-AGC CTC GTC CCG TAG ACA AAA-3'<br>5'-TTT TGG CTC CAC CCC TTC A-3'	367
Gapdh inner	5'-TGA AGG TCG GTG TGA ACG AAT T-3'<br>5'- GCT TTC TCC ATG GTG GTG AAG A-3'	313

Table S4. Primer sets for DNA methylation analysis

Gene	Primer Sequence	Size of PCR products (bp)
Nfat1 methylation	5'- TCG TTT AGG TTA GAT CGG GC-3'<br>5'- AAC GAC GCG AAC TTC CTA CT-3'	122
Nfat1 *non*-methylation	5'- TTG TTG TTT AGG TTA GAT TGG GT-3'<br>5'- AAC AAC ACA AAC TTC CTA CTC AA-3'	122
Nfat1 BSP	5'- TTT GGT ATG AGT TAT AGT TGT GGG-3'<br>5'- TTC CTA CTC AAA ACA CCT ATT ACA AC-3'	278
Nfat1 (MeDIP- and hMeDIP PCR)	5'- GTG GCT GCT ATA TGC TGG GT-3'<br>5'-TGG CTC ATG CCA AAG TGT CT-3'	99

Table S5. Primer sets for ChIP-PCR

Gene	Primer Sequence	Size of PCR products (bp)
Itgam	5'- GTC AGC GCT TAG TGG CAA AC-3'<br>5'- GTG CTC CCT TCA GCA CTC AT-3'	152
Tnf	5'- ACC GCA GTC AAG ATA TGG CA-3'<br>5'- ATT CAC GGA CCT CAC AAG CC-3'	117
Il-1b	5'- CCT GGC AGG GCA GGA AAG-3'<br>5'- TGG AAG CAA GCC TAT GCA GT-3'	199
c-Myc	5'-ATA CGT GGC AGT GAG TTG CT-3'<br>5'-GAG GGT GAT CAA CCG CAG AT-3'	88
